# Systemic prime mucosal boost significantly increases protective efficacy of bivalent RSV influenza viral vectored vaccine

**DOI:** 10.1038/s41541-024-00912-1

**Published:** 2024-06-26

**Authors:** Cameron Bissett, Sandra Belij-Rammerstorfer, Marta Ulaszewska, Holly Smith, Reshma Kailath, Susan Morris, Claire Powers, Sarah Sebastian, Hannah R. Sharpe, Elizabeth R. Allen, Ziyin Wang, Robert F. Cunliffe, Hadijatou J. Sallah, Alexandra J. Spencer, Sarah Gilbert, John S. Tregoning, Teresa Lambe

**Affiliations:** 1 Oxford Vaccine Group, Department of Paediatrics, University of Oxford, Oxford, UK; 2 Pandemic Sciences Institute, Nuffield Department of Medicine, University of Oxford, Oxford, UK; 3 The Jenner Institute, Nuffield Department of Medicine, University of Oxford, Oxford, UK; 4 Department of Infectious Disease, Imperial College London, London, UK; 5 School of Biomedical Sciences and Pharmacy, University of Newcastle, Newcastle, Australia

**Keywords:** Vaccines, Adaptive immunity

## Abstract

Although licensed vaccines against influenza virus have been successful in reducing pathogen-mediated disease, they have been less effective at preventing viral infection of the airways and current seasonal updates to influenza vaccines do not always successfully accommodate viral drift. Most licensed influenza and recently licensed RSV vaccines are administered via the intramuscular route. Alternative immunisation strategies, such as intranasal vaccinations, and “prime-pull” regimens, may deliver a more sterilising form of protection against respiratory viruses. A bivalent ChAdOx1-based vaccine (ChAdOx1-NP + M1-RSVF) encoding conserved nucleoprotein and matrix 1 proteins from influenza A virus and a modified pre-fusion stabilised RSV A F protein, was designed, developed and tested in preclinical animal models. The aim was to induce broad, cross-protective tissue-resident T cells against heterotypic influenza viruses and neutralising antibodies against RSV in the respiratory mucosa and systemically. When administered via an intramuscular prime-intranasal boost (IM-IN) regimen in mice, superior protection was generated against challenge with either RSV A, Influenza A H3N2 or H1N1. These results support further clinical development of a pan influenza & RSV vaccine administered in a prime-pull regimen.

## Introduction

The route of vaccination is an important consideration when designing vaccines against respiratory pathogens. Upper respiratory tract (URT) immunity to respiratory syncytial virus (RSV) and influenza virus has been shown to protect against viral infection and disease^1–5^. Tissue-resident memory T and B cells (T_RM_ and B_RM_), as well as neutralising IgA within the upper respiratory tract, contribute to rapid response and control of viral infection at the site of viral entry^6^. Standard intramuscular (IM) administration, used for most licensed influenza vaccines and the recently licensed RSV vaccines, is known to be protective against disease. However, IM vaccination is largely ineffective at stimulating the mucosal immune compartment^7–9^. Both clinical and preclinical studies have shown that delivering influenza and RSV vaccines via mucosal routes better induces mucosal immunity and correlates well with protection^10–16^.

Considerable progress has been made in determining the best antigenic candidates for RSV and influenza vaccines. RSV’s fusion protein (RSVF) is conserved across viral subtypes, and has a critical role in mediating virus-host membrane fusion^17,18^. RSVF is metastable in its prefusion form and can spontaneously convert into a more stable postfusion form, which conceals epitopes targeted by potently neutralising antibodies (NAbs)^19–21^. Stabilised prefusion forms of RSVF protein have been shown to better induce NAbs compared with postfusion forms of RSVF^19,22^. Modified, stabilised RSVF antigen is now used in a number of vaccines that have demonstrated efficacy against RSV respiratory tract infection and/or disease, including vaccines from GSK, Pfizer and Moderna^23^.

The ability of traditional seasonal influenza candidate vaccines to induce hemagglutinin-inhibiting antibodies as measured via hemagglutination inhibition assay (HAI) is a determining factor for their licensure^24^. Predictions of the identity of the next-season circulating strain(s), and subsequent updates of last-season vaccines, are completed such that protective NAbs should be induced by the vaccine; this process is costly and not always effective as mismatch frequently occurs^25^. Alternative vaccine approaches have been tested at the preclinical and clinical stage; some of which aim to induce different forms of protection, and that deviate from HAI-based strategies. One approach focuses on conserved antigens, e.g., nucleoprotein (NP) and matrix 1 (M1), with the aim of inducing CD8^+^ T cell populations that are broadly reactive to different influenza A subtypes^26,27^. By facilitating rapid clearance of the virus within the URT, NP- and M1-specific T cells are thought to limit virus infection and lessen disease severity^10,27^.

A suitable vector for the delivery and expression of influenza NP and M1 T cell antigens, and RSV F B cell antigen, is adenovirus ChAdOx1. The safety profile and immunogenicity of this platform has been well-studied for vaccines against various pathogens, including SARS-CoV-2^28–32^. IM immunisation with ChAdOx1 has been used in global vaccination schemes, and this vaccine technology can be administered safety via the mucosal intranasal (IN) route^7,33^. Additionally, it can be modified to express multiple antigenic sequences facilitating immunity to multiple pathogens through one vaccine dose.

This study aimed to assess the immunogenicity and protective capacity of ChAdOx1-NP + M1-RSVF, a bivalent ChAdOx1 viral vector vaccine, against influenza virus and RSV infection and disease. ChAdOx1-NP + M1-RSVF incorporates the pre-F RSV antigen and influenza nucleoprotein and matrix 1 antigens. We specifically investigated the impact of mucosal and systemic vaccine delivery on the immunogenicity and protective response against challenge with virus RSV A, as well as influenza A viruses H3N2 and H1N1. We demonstrate that an intramuscular prime-intranasal boost combination gave the best protection against infection with all three viruses.

## Results

### IM-IN vaccination elicits superior antigen-specific antibody response in the blood and respiratory mucosa

ChAdOx1-NP + M1-RSVF was constructed by inserting antigenic sequences within the ChAdOx1 backbone. A pre-fusion-stabilised RSV A2-F (DS2) sequence was inserted at the E4-deleted gene site, and a sequence encoding H3N2 NP and M1 proteins, attached to each other by a flexible linker, were inserted at the E1-deleted gene site.

ChAdOx1 vaccines previously tested at preclinical and clinical level can induce protection against infection and disease when administered in a single-dose or two-dose, prime-boost regimen. We tested a variety of prime-only and prime-boost regimens involving IM and/or IN routes of vaccination, to determine which regimens induced both systemic, and respiratory mucosal immune responses to RSV and influenza A antigens and optimal immunogenicity. An outbred (CD-1) mouse strain was selected, such that immune responses to vaccination better represented an outbred population as is similar to humans.

**Fig. 1:**
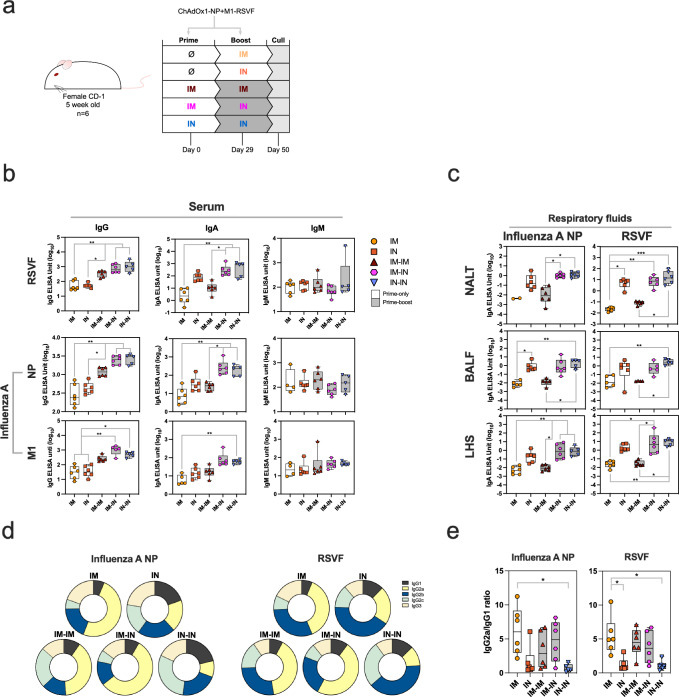
Humoral immunogenicity of ChAdOx1-NP + M1-RSVF. **a** Vaccination schematic for the assessment of the immunogenicity of ChAdOx1-NP + M1-RSVF administered through different regimens in outbred, 5-week-old, CD-1 mice (*n* = 6). Mice were culled on day 50, with sera, lungs, spleens, nasal-associated lymphoid tissue (NALT) and bronchioalveolar lavage fluid (BALF) harvested. **b** IgG, IgA and IgM responses against RSV-F, influenza A NP and M1 (H1N1) in sera three weeks post-final vaccination, measured by ELISA. Values are displayed as ELISA units (EUs) (log_10_). Individual mouse values are represented as symbols. Values were analysed using non-parametric Kruskal-Wallis tests to assess for statistically significant differences between groups, which are then expressed as *p* values (*=*p* < 0.05, **=*p* < 0.01, ***=*p* < 0.001). **c** Levels of IgA specific to influenza A NP (H1N1) and RSVF in NALT, BALF and lung homogenate supernatant (LHS) collected from mice three weeks post-final vaccination as measured by ELISAs (*=*p* < 0.05, **=*p* < 0.01, ***=*p* < 0.001). **d** Relative levels of anti-influenza A NP (H1N1) and anti-RSVF IgG subclasses in serum (IgG1, IgG2a, IgG2b, IgG2c and IgG3), as measured by tIgG-normalised indirect ELISA. Relative IgG subclass levels following each regimen are presented as individual doughnut charts, with each section of the doughnut representing the median IgG subclass OD_405nm_ response. Bar charts with individual sample responses per subclass per regimen are present in Supplementary Fig. 3a. **e** Serum antigen-specific IgG2a to IgG1 subclass ratios (IgG2a OD_405nm_/IgG1 OD_405nm_). For all boxplots, whisker endings represent upper and lower extremes, the box bounds represent upper and lower quartiles, respectively, and the central line represents the group median.

Three weeks after the final vaccination the levels of IgG, IgA and IgM specific to H1N1-NP, H1N1-M1, H3N2-NP, H3N2-M1 and RSV A2-F antigens were measured via ELISA in the serum of vaccinated mice (Fig. 1a, b and Supplementary Fig. 1a). Serum IgG titres against all antigens were detected (Fig. 1b). All prime-boost regimens induced higher IgG levels compared with prime-only regimens (Fig. 1b). Specifically, IM-IN and IN-IN regimens elicited significantly higher IgG titres than prime-only regimens; this finding was consistent across all three antigens (RSV A2-F, H1N1-NP and H1N1-M1) (Fig. 1b and Supplementary Table 1a). IM-IN and IN-IN regimens also elicited between 2- and 5-fold higher log_10_ IgG ELISA units (EUs) than in IM-IM mice, however, these differences were not significant. IM-IM vaccination did not elicit significantly higher IgG titres in sera compared with prime-only groups. Increased antigen-specific IgA levels in serum were observed in IM-IN and IN-IN compared with IM and IM-IM, specific against RSV A2-F, H1N1-NP and H1N1-M1 (Fig. 1b and Supplementary Table 1a).

To assess mucosal antibody responses, respiratory fluid IgA specific to H1N1-NP (as the representative influenza A antigen) and RSV A2-F were tested (Fig. 1c and Supplementary Fig. 2a and b). Higher levels of H1N1 NP- and RSV A2 F-specific IgA in IM-IN and IN-IN were detected within nasal-associated lymphoid tissue (NALT), broncho-alveolar lavage fluid (BALF) and lung homogenate supernatant (LHS), compared with levels following IM and IM-IM vaccination (Fig. 1c and Supplementary Table 1b). The IN-IN regimen elicited the highest titres of IgA in NALT, BALF and LHS. IM-IN-vaccinated groups had IgA titres comparable to the IN-IN regimen in NALT, BALF and LHS.

To assess if regimens elicited Th1 (increased relative IgG2a) or Th2 (increased relative IgG1) responses, the relative abundances of IgG subclasses specific to NP and RSVF antigens in serum were then assessed. IM, IM-IM and IM-IN regimens elicited high relative levels of IgG2a among subclasses, suggesting a Th1-type response (Fig. 1d and e)^34^. IN and IN-IN groups, however, did not elicit clear Th1-type IgG subclass responses, with reduced relative levels of IgG2a, and either balanced levels across all five subclasses (H1N1 NP-specific) or elevated IgG2b (RSVF-specific) (Fig. 1d).

In summary, IM-IN and IN-IN regimens induced higher systemic and respiratory mucosal antibody responses against viral antigens compared with other regimens tested. However, IM-IN-vaccinated mice had a clear Th1-type systemic IgG subclass profile, whereas IN-IN-vaccinated mice possessed a mixed IgG subclass profile and IgG2b-dominant profile, for influenza NP-specific and RSVF-specific IgG, respectively.

### IM-IN vaccination prompts strong T-cell response and the generation of T_RM_ in the lungs

**Fig. 2:**
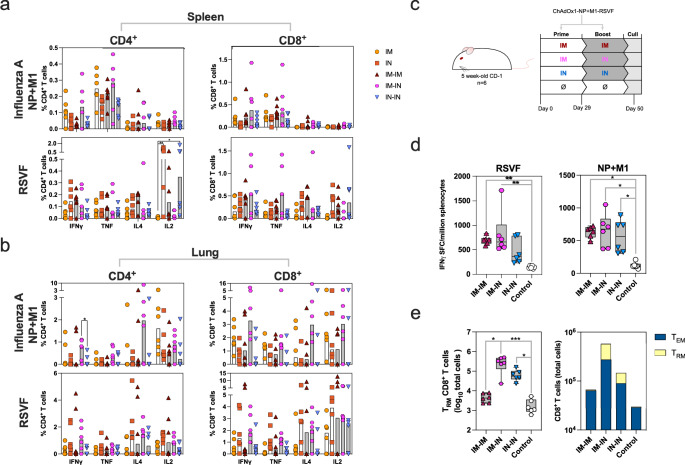
Cellular immunogenicity of ChAdOx1-NP + M1-RSVF. **a** Cytokine responses in CD4^+^ and CD8^+^ splenocytes that were separately stimulated with influenza A (H1N1) NP + M1- and RSVF-spanning peptides; % cytokine^+^ T cells were determined through intracellular staining. Basal frequencies of cytokine^+^ T cells (unstimulated sample) were subtracted from stimulated sample frequencies. Median responses in each group are displayed as the top lines of bars on graphs on the left-hand side of the figure, with symbols representing individual mice, grey-shaded bars prime-boost regimens and white bars prime-only regimens. **b** Cytokine responses in CD4^+^ and CD8^+^ lung cells harvested from mice, separately stimulated with influenza A (H1N1) NP + M1- and RSVF-spanning peptides. **c** Vaccination schematic for the continued assessment of the cellular immunogenicity of ChAdOx1-NP + M1-RSVF. **d** IFNγ responses in splenocytes stimulated with NP + M1- and RSVF-spanning peptides (IFNγ spot-forming cells (SFCs)/million splenocytes), as measured by IFNγ enzyme-linked immunosorbent spot (ELISpot) assay. **e** Total counts of CD8^+^ T_RM_ cells (left), as well as relative counts of CD8^+^ T_EM_ and T_RM_ cells (right) in lungs post-vaccination (all non-antigen-specific). T_RM_ cells were defined as CD8^+^CD44^+^CD62L^-^CD103^+^CD69^+^, and negative for intravenous (IV) circulatory CD3^+^ T cell stain. T_EM_ cells were defined as CD8^+^CD44^+^CD62L^-^CD127^+^, and negative for IV circulatory CD3^+^ T cell stain. Group differences of data in (**a**), (**b**), (**d**) and (**e**) were analysed using non-parametric Kruskal-Wallis tests (*=*p* < 0.05, **=*p* < 0.01, ***=*p* < 0.001). For all boxplots, whisker endings represent upper and lower extremes, the box bounds represent upper and lower quartiles, respectively, and the central line represents the group median.

T-cell responses were measured in the spleen and lungs to determine the systemic and respiratory cellular responses, respectively. Antigen peptide stimulation and subsequent intracellular staining (ICS) of cytokines IFNγ, TNF, IL-4 and IL-2 was performed to determine the frequency of CD8^+^ and CD4^+^ T cells expressing each cytokine, and subsequent T cell phenotype (Fig. 2a).

Splenocytes were stimulated with NP and M1 peptides, and following IM-IN vaccination, CD4^+^s demonstrated a trend of highest levels of IFNγ and TNF production and minimal IL-4 and IL-2, compared with other regimens (Fig. 2a). RSVF-stimulated splenocytes of IN- and IN-IN-vaccinated mice had strikingly higher levels of IL-2 staining in CD4^+^ cells compared with IM and IM-IM regimen CD4^+^ cells (IN > IM and IN-IN > IM, *P* = 0.0082 and *P* = 0.0297, respectively). Relative levels of staining of other cytokines IFNγ, TNF and IL-4, however, remained statistically comparable between regimens following RSVF peptide stimulation.

Following NP and M1 peptide stimulation, CD8^+^ splenocytes had a trend across all regimens of minimal IL-4 and IL-2 expression, and higher levels of IFNγ and TNF, which were highest following the IM-IN regimen (Fig. 2a). Following RSVF peptide stimulation, CD8^+^ splenocytes following IM-IN mirrored this trend. Trends in expression following other regimens (IM, IN, IM-IM and IN-IN) were not detected.

Cytokine-positive responses were detected in CD4^+^ and CD8^+^ lung cells to varying degrees (Fig. 2b). Lung CD4^+^s and CD8^+^s of IM-IN-vaccinated mice expressed the highest levels of IFNγ, TNF and IL-4 following NP- and M1-peptide stimulation, although these differences were not statistically significant (Fig. 2b). Notably, lung cell cytokine responses following IN-IN vaccination were generally low compared with other regimens. Overall, measured lung CD4^+^ and CD8^+^ T cell cytokine responses had a mixed profile post-peptide-stimulation.

Splenocyte IFNγ release following peptide stimulation was also assessed through IFNγ ELISpot assay, in a repeated experiment focussing on IM-IM, IM-IN and IN-IN regimens (Fig. 2c and d). Following RSVF peptide stimulation, IM-IM- and IM-IN-vaccinated mouse splenocytes released comparable levels of IFNγ that were statistically higher than control, unvaccinated mice (*P* = 0.0057 and *P* = 0.0065, IM-IM>unvaccinated control and IM-IN>unvaccinated control, respectively) (Fig. 2d). Following NP + M1 peptide stimulation, IM-IM, IM-IN and IN-IN mouse group splenocytes all released higher levels of IFNγ compared with unvaccinated control (*P* = 0.0256, *P* = 0.0100, *P* = 0.0291, IM-IM>unvaccinated control, IM-IN>unvaccinated control, and IN-IN>unvaccinated control, respectively) (Fig. 2d).

Total numbers of lung tissue-resident memory T cells (T_RM_) were measured via antibody staining and flow cytometry. T_RM_ were defined as expressing a CD3^IV^^-^CD69^+^CD103^+^CD62L^-^CD44^+^ phenotype, where anti-CD3 antibody was intravenously (IV) injected into mice to exclude all circulatory T cells (Fig. 2e). Lung CD8^+^ T_RM_ counts were highest following IM-IN vaccination (IM-IN > IM-IM, *P* = 0.0197 and IM-IN>unvaccinated control, *P* = 0.0005). IN-IN vaccinated mice had T_RM_ counts lower than IM-IN, but that were still higher than unvaccinated control mice (IN-IN>unvaccinated control, *P* = 0.0197). Additionally, T_EM_ (CD3^IV^^-^CD44^+^CD62L^-^CD127^+^) counts were highest following IM-IN vaccination (Fig. 2e).

Overall magnitudes of T cell cytokine responses tended to be higher in IM-IN-vaccinated groups both systemically in the spleen and locally in the lung. In the spleen, these responses were generally IFNγ and TNF biased, whereas in the lungs a mixed cytokine profile was detected. IM-IM- and IM-IN-vaccinated mouse splenocytes also released high levels of IFNγ as measured by ELISpot assay. The IM-IN regimen was able to potentiate higher counts of T_RM_ and T_EM_ in the lungs compared with other regimens. A summary of the magnitude of the immune response following the reach regimen is represented through a normalised heatmap in Supplementary Fig. 4a.

### IM-IN and IN-IN immunisation best protect against RSV infection and disease

**Fig. 3:**
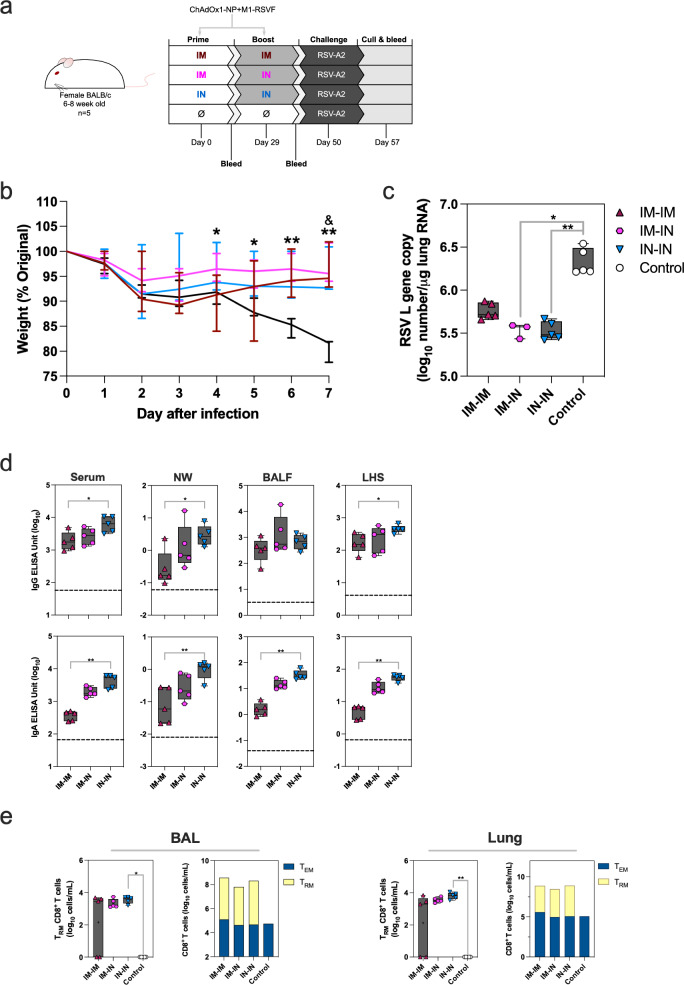
Challenging ChAdOx1-NP + M1-RSVF-vaccinated mice with RSV. **a** Vaccination and challenge schematic for the assessment of the protective capacity of ChAdOx1-NP + M1-RSVF against RSV-A2 infection. Mice were prime-boost-vaccinated, then challenged with RSV-A2, and culled 7 days later with tissues and fluids harvested. Blood sampling was performed 4 weeks post-prime and 3 weeks post-boost. **b** Weight change in mice over time post-challenge, measured as % of pre-challenge weight. At each timepoint, lines represent group medians, and bars represent group bodyweight ranges. Significant differences between IM-IN and control groups are represented with *, between IM-IM and control as & (* or & =*p* < 0.05, **=*p* < 0.01). **c** Viral load in lungs 7 days post-challenge, measured as number of RSV L gene copies/μg lung RNA (log_10_). **d** RSVF-specific IgG and IgA levels in serum, NWs, BALF and LHS post-challenge, measured by ELISA. Median negative control values are displayed as dashed lines. **e** Levels of antigen-specific CD8^+^ T_RM_, and relative levels of antigen-specific CD8^+^ T_EM_ and T_RM_, in BAL and lungs post-challenge. T_RM_ and T_EM_ cells were defined as CD3^+^CD8^+^CD44^+^CD62L^-^CD103^+^CD69^+^, and CD3^+^CD8^+^CD44^+^CD62L^-^, respectively, and positive for RSV pentamer H-2Kd KYKNVTEL. In boxplots, a “+” symbol represents the group mean. Two mice from the IM-IM-vaccinated group did not have detectable BAL or lung T_RM_. Group differences for all data were analysed using non-parametric Kruskal-Wallis tests (*=*p* < 0.05, **=*p* < 0.01). For all figure boxplots, whisker endings represent upper and lower extremes, the box bounds represent upper and lower quartiles, respectively, and the central line represents the group median.

The level of protection against RSV following IM-IM, IM-IN or IN-IN ChAdOx1-NP + M1-RSVF vaccination regimen was assessed via challenge. 5-week-old BALB/c mice were vaccinated, and then challenged IN with 7.7 × 10^5^ PFU RSV-A2 21 days-post boost (Fig. 3a).

Weight loss was measured daily in mice post-challenge as a measure of deteriorating health and disease. All vaccinated mice, irrespective of regimen, followed a trend of less weight loss compared with unvaccinated, then challenged, control mice (Fig. 3b). Weight loss between days 4 and 7 post-infection within the IM-IN-vaccinated group was significantly less when compared with the control group (Fig. 3b, Supplementary Table 2a and Supplementary Fig. 5a). No significant differences in weight loss were measured between immunisation regimens. To assess the degree of viral replication in the respiratory tract, lung RSV viral loads were measured after mice were culled on day 7. IM-IN and IN-IN-vaccinated mice had statistically lower viral loads within lungs compared with unvaccinated, challenged controls (*P* = 0.0166 and *P* = 0.0055, IM-IN>unvaccinated control and IN-IN>unvaccinated control, respectively) (Fig. 3c).

Humoral antibody and cellular immune responses systemically and in the respiratory tract were assessed on day 7 of the challenge. IgG and IgA antibodies against RSV A2-F were detected above the control baseline in all regimens in serum 7 days post-challenge (Fig. 3d and Supplementary Fig. 6a). Within nasal washes (NWs), BALF and LHS, IN-IN-vaccinated mice had significantly higher titres of IgG and IgA compared with IM-IM-vaccinated mice post-challenge, whilst IM-IN-vaccinated mice had intermediate values between such regimens (Supplementary Table 3a).

Total counts of T_RM_ (defined as CD3^+^CD69^+^CD103^+^CD62L^-^CD44^+^, and H-2Kd KYKNVTEL^+^) in BAL and lungs were measured. Overall counts of BAL and lung CD8^+^ T_RM_ within all vaccinated, challenged groups were higher than unvaccinated, challenged control sample levels (Fig. 3e). Levels of tissue-resident CD8^+^ T cells of BAL and lung were of statistically insignificant difference between regimens, and only IN-IN-vaccinated mice demonstrated statistically higher levels compared with unvaccinated control (*P* = 0.0134 in BAL cells and *P* = 0.0028 in lung cells) (Fig. 3e). Nevertheless, IM-IN and IN-IN regimens had BAL and lung T_RM_ levels of much smaller range compared with IM-IM-vaccinated mice; IM-IM vaccinated mice had larger ranges of values, and two mice with T_RM_ levels below the limit of detection. All vaccinated, RSV-challenged mice contained numbers of T_EM_ CD8^+^ T cells similar level to naïve, challenged mice in BAL and lungs.

All vaccinated animals were protected from overt disease with IM-IN and IN-IN immunised mice having a significant reduction of lung viral load following the RSV challenge.

### IM-IN and IN-IN immunisation best protect against H3N2 infection and disease

**Fig. 4:**
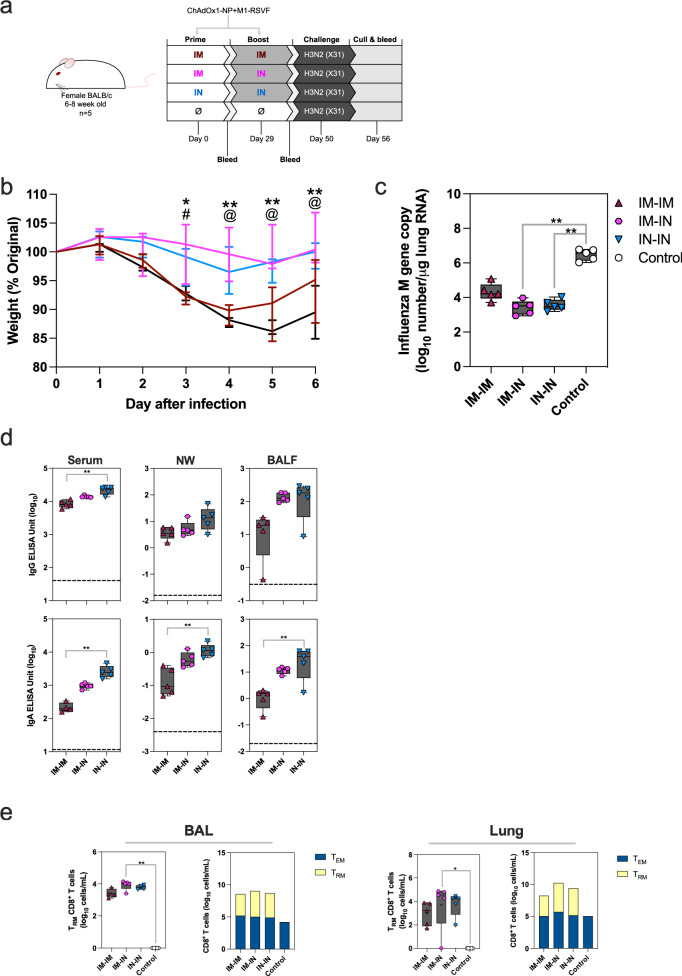
Challenging ChAdOx1-NP + M1-RSVF-vaccinated mice with H3N2. **a** Vaccination and challenge schematic for the assessment of the protective capacity of ChAdOx1-NP + M1-RSVF against X31 (H3N2) infection and subsequent disease in mice. Mice were prime-boost-vaccinated, then challenged with X31, and culled 6 days later with tissues and fluids harvested. Blood sampling was performed 4 weeks post-prime and 3 weeks post-boost. **b** Weight change in mice over time post-challenge, measured as % of pre-challenge weight. Significant differences at timepoints between IM-IN and control mouse groups are represented with *, between IM-IN and IM-IM as # and between IN-IN and unvaccinated as @ (*, @ or # =*p* < 0.05, **=*p* < 0.01). **c** Viral load in lungs 6 days post-challenge (M gene copies/μg lung RNA (log_10_)). **d** H3N2 NP-specific IgG and IgA levels in serum, NWs, BALF and LHS post-challenge, as measured by ELISA (log_10_ EU). Median negative control values are displayed as dashed lines. **e** Levels of antigen-specific CD8^+^ T_RM_ cells, and relative levels of antigen-specific CD8^+^ T_EM_ and T_RM_, in BAL and lungs post-challenge. T_RM_ and T_EM_ cells were defined as CD3^+^CD8^+^CD44^+^CD62L^-^CD103^+^CD69^+^, and CD3^+^CD8^+^CD44^+^CD62L^-^, respectively, and positive for influenza pentamer H-2Kd TYQRTALV. In boxplots, a “+” symbol represents the group mean. One mouse in group IM-IN did not have detectable Lung T_RM_. Group differences for all data were analysed using non-parametric Kruskal-Wallis tests (*=*p* < 0.05, **=*p* < 0.01). For all boxplots, whisker endings represent upper and lower extremes, the box bounds represent upper and lower quartiles, respectively, and the central line represents the group median.

The level of protection against influenza A subtype H3N2 following IM-IM, IM-IN or IN-IN ChAdOx1-NP + M1-RSVF vaccination regimen was assessed via challenge. BALB/c mice were vaccinated, and then challenged IN with 2 × 10^5^ PFU influenza A X-31 21 days-post boost (Fig. 4a).

All vaccinated groups lost less weight than controls after the challenge. Mice vaccinated IM-IN or IN-IN, lost the least weight upon challenge (*P* = 0.0066 and *P* = 0.02332, IM-IN>unvaccinated control and IN-IN>unvaccinated control, respectively) (Fig. 4b, Supplementary Fig. 5b and Table 2b). IM-IM-vaccinated mice lost more weight, mirroring weight loss observed in unvaccinated, challenged control mice until day 4. Following day 4, IM-IM-vaccinated mouse weights began to recover until cull at day 6 post-challenge.

All vaccinated, H3N2-challenged mice had less viral load in lungs (measured as M gene copy number/μg lung RNA) compared with unvaccinated, challenged control mice (Fig. 4c). Mice vaccinated with IM-IN and IN-IN regimens had statistically lower viral loads compared with unvaccinated control mice (*P* = 0.0037 and *P* = 0.0097, IM-IN>unvaccinated control and IN-IN>unvaccinated control, respectively), and viral loads were of a similar level across IM-IN and IN-IN regimens.

Humoral antibody and cellular immune responses systemically and in the respiratory tract were assessed on day 6 of the challenge. Serum IgG and IgA antibodies against H3N2-NP were detected above baseline in all regimens (Fig. 4d and Supplementary Fig. 6b). Serum and airway IgG and IgA were significantly higher following IN-IN vaccination and challenge, than IM-IM vaccination and challenge, whilst IM-IN-vaccinated mice again had intermediate titres (Supplementary Table 3b).

CD8^+^ T_RM_ cells (defined as CD3^+^CD69^+^CD103^+^CD62L^-^CD44^+^, and H-2Kd TYQRTRALV^+^) were detected in BAL and lungs above the level in control, challenged animals in all vaccination regimens (Fig. 4e). In both BAL and lungs, IM-IN-vaccinated, challenged mice had the highest median T_RM_ CD8^+^ T cell frequencies across immunisation regimens, and were significantly higher than in challenged control group (*P* = 0.0020 and *P* = 0.0101 for BAL and lung cells, respectively). One IM-IN-vaccinated mouse failed to generate detectable lung T_RM_ CD8^+^ T cells following the challenge. Following all regimens, T_EM_ cells could be detected in BAL and lungs (Fig. 4e).

All vaccinated animals were protected from overt disease with viral titres being lower following IM-IN and IN-IN regimen than IM-IM and higher frequencies of a T_RM_ population in the IM-IN-vaccinated groups post-H3N2 challenge.

### IM-IN immunisation provides some protection against H1N1 infection

**Fig. 5:**
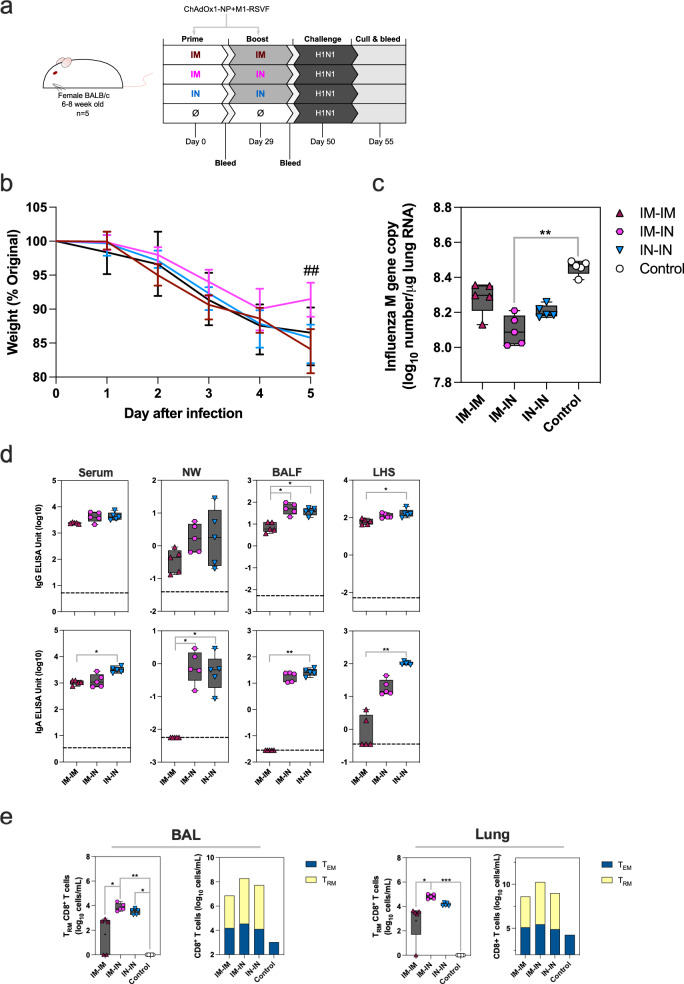
Challenging ChAdOx1-NP + M1-RSVF-vaccinated mice with H1N1. **a** Vaccination and challenge schematic for the assessment of the protective capacity of ChAdOx1-NP + M1-RSVF against H1N1 infection and subsequent disease in mice. Mice were prime-boost-vaccinated, then challenged with H1N1, and culled 5 days later with tissues and fluids harvested. Blood sampling was performed 4 weeks post-priming and 3 weeks post-boosting. **b** Weight change in mice over time post-challenge, as measured by % of pre-challenge weight. The significant difference between IM-IN and IM-IM mouse groups is represented with ## (##=*p* < 0.01). **c** Viral load in lungs 5 days post-challenge (number of M gene copies/μg lung RNA (log_10_)). **d** H1N1 NP-specific IgG and IgA levels in serum, NWs, BALF and LHS post-challenge, as measured ELISA (log_10_ EU). Median control values are displayed as dashed lines on graphs. **e** Levels of antigen-specific CD8^+^ T_RM_ cells^,^ and relative levels of antigen-specific CD8^+^ T_EM_ and T_RM_, in BAL and lungs post-challenge. T_RM_ and T_EM_ cells were defined as CD3^+^CD8^+^CD44^+^CD62L^-^CD103^+^CD69^+^, and CD3^+^CD8^+^CD44^+^CD62L^-^, respectively, and positive for influenza pentamer H-2Kd TYQRTALV. In boxplots, a “+” symbol represents the group mean. Two mice in group IM-IM did not have detectable BAL T_RM_, and one mouse in group IM-IM did not have detectable Lung T_RM_. Group differences for all data were analysed using non-parametric Kruskal-Wallis tests (*=*p* < 0.05, **=p < 0.01, ***=*p* < 0.001). For all boxplots, whisker endings represent upper and lower extremes, the box bounds represent upper and lower quartiles, respectively, and the central line represents the group median.

The level of protection against influenza A strain H1N1 following IM-IM, IM-IN or IN-IN ChAdOx1-NP + M1-RSVF vaccination regimen was assessed through challenge. 5-week-old BALB/c mice were vaccinated, and then challenged IN with 2.1 × 10^5^ PFU influenza A H1N1 21 days-post boost (Fig. 5a).

Weight changes in mice following challenge were similar among vaccinated and unvaccinated control groups following challenge until days 2-5, when weight loss in IM-IN-vaccinated mice halted and their weight began to increase after day 4, unlike other groups (Fig. 5b). This elevated weight in IM-IN mice on day 5 was statistically higher than IM-IM-vaccinated mice (IM-IN > IM-IM *P* = 0.0066).

Viral loads in lungs (measured by M gene copy number/μg lung RNA) were all lower in vaccinated groups, however, only mice vaccinated IM-IN had statistically significant lower viral load compared with unvaccinated, challenged control mice (*P* = 0.0011, IM-IN vs. unvaccinated control); IM-IN-vaccinated mice had the lowest detectable viral loads among all vaccinated regimen mouse groups (Fig. 5c).

Humoral antibody and cellular immune responses systemically and in the respiratory tract were assessed on day 5 of the challenge. IgG and IgA antibodies against H1N1-NP were detected above the control baseline in all regimens in serum 5 days post-challenge (Fig. 5d and Supplementary Fig. 6c). Within the airways of IM-IN- and IN-IN-vaccinated mice, higher IgG and IgA titres were detected compared with IM-IM vaccinated mice post-challenge (Supplementary Table 3c).

Within BAL and Lungs, IM-IN-vaccinated, challenged mice had more CD8^+^ T_RM_ cells (defined identically to that of H3N2 challenge), and the total number were statistically higher than measured in IM-IM-vaccinated mice *(P* = 0.0379 and *P* = 0.0267 for BAL and lung cells, respectively) (Fig. 5e). All regimens induced measurable numbers of T_EM_ cells compared with unvaccinated, challenged control mice. IM-IN vaccination, however, induced the greatest numbers of T_EM_ cells compared with other regimens.

IM-IN immunised mice demonstrated greater protection compared with other regimens, though not sterilising; increased weight after day 4 of infection and lower viral loads were paired with distinctively higher levels of CD8^+^ T_RM_ and T_EM_ locally in BAL and lungs.

## Discussion

RSV and influenza A alongside SARS-CoV-2, have driven the “tridemic”; the cocirculation of all three respiratory viruses has resulted in a massive global disease burden. RSV and influenza A in particular, disproportionately impact children and the elderly. Recently, RSV vaccine candidates and a long-lasting antibody therapeutic (Nirsevimab) have been licensed, and vaccines against influenza are used in annual vaccination regimens^23^. Licensed RSV and influenza vaccines are mostly administered intramuscularly; a safe route associated with systemic immunity, and effective at eliminating severe disease. However, the IM route of vaccination has limited capacity to induce respiratory mucosal immunity, which has been associated with the control of respiratory infection^35,36^. A respiratory mucosal route of vaccination capable of inducing durable RSVF-specific memory B cells and neutralising antibodies at the site of viral entry will provide protection and reduce levels of infection^2,37^. Additionally, T_RM_ induced upon mucosal vaccination resting within the lung parenchyma and airway mucosa, capable of rapid activation upon influenza A infection, are expected to largely enhance the control of virus infection^38–40^.

As SARS-CoV-2, RSV and influenza persist globally, cost-effective, two-in-one bivalent vaccines, capable of inducing simultaneous respiratory and systemic immunity against RSV and influenza present an attractive strategy. In this study, a bivalent vaccine ChAdOx1-NP + M1-RSVF was designed and tested in mice. Whilst other animal models such as ferret for influenza virus and cotton rat for RSV may recapitulate aspects of the human immune response, the mouse model is well established for these types of immunogenicity and challenge study^41,42^; it can be used to discern and predict which regimens/vaccines may be more immunogenic and protective over others. It also has considerable overlaps with human infection, especially severe disease following infection^41–49^. Assessment of the immunogenicity and protection conferred after vaccination with ChAdOx1-NP + M1-RSVF supports further clinical development. Our work demonstrated that the route of vaccination and regimen have a strong influence on the nature of immune response and the extent of protection provided by ChAdOx1-NP + M1-RSVF. IM-IN-vaccinated mice generally demonstrated higher magnitudes of cellular and humoral responses, and a favourable Th1-skewed IgG subclass response was observed. The IM-IN regimen also demonstrated greater overall protection against infection over other homologous route regimens; the extent to which IM-IN vaccination offered greater protection, however, varied depending on the virus used in the challenge. Furthermore, the protection provided by IM-IN vaccination was likely via different mechanisms depending on whether RSV or influenza virus.

Greater numbers of CD8^+^ T_RM_ were noted in the lungs and BAL of IM-IN-vaccinated mice challenged with H3N2 and H1N1 influenza A strains, compared with those challenged with RSV. Preclinical studies have used conserved NP and M1 antigens as targets in their influenza vaccines for the generation of cross-protective T cells^27,50–52^. Some of these studies used NP and M1 antigens encoded in ChAdOx-based vectors and detected distinct differences in responses depending on the route of vaccination; aerosol vaccination of pre-exposed pigs with ChAdOx2-NP + M1-NA boosted mucosal immunity, and IM vaccination solely peripheral blood immunity^51^. A human trial using a MVA vectored, NP + M1-encoding vaccine concluded no advantageous protection over the control group when administered IM in individuals vaccinated 28 days prior with standard quadrivalent influenza vaccine (QIV)^53^. Here, it was suggested that alternative routes of vaccination such as aerosol or IN be considered for enhanced efficacy of vaccine^53^. It is likely that lung-resident cross-reactive T_RM_ cells are important for greater control over influenza infection than what is provided through systemic vaccination^38–40^.

In addition to T_RM_ cells, ChAdOx1-NP + M1-RSVF elicited antibody responses against internal influenza A proteins NP and M1. Unlike NP- and M1-reactive CD8^+^ T cells, it is unclear what role anti-NP and M1 antibodies may contribute, if any, to protection against influenza infection and disease in humans. It has been suggested that anti-NP IgG antibodies that are capable of engaging effector cells via Fc-receptor-Fc binding may contribute to protection; such antibodies have been shown to be common in healthy and influenza-infected adults^54^. However, various small animal model studies have presented conflicting data in support of and against a protective role of anti-NP and M1 influenza protein antibodies^55–58^.

IM-IN-vaccinated mice were less protected against H1N1 (A/California/7/2009) infection and disease, compared with when they were challenged with H3N2 (X31), despite both strains’ NP and M1 sequences being conserved to a similarly high degree to that encoded in the vaccine. Here, T cell immunity and potentially anti-NP and M1 antibodies were likely not adequate for sterilising protection against A/California/7/2009, which has shown to be more virulent in mouse infection models, compared with X31^59^.

All ChAdOx1-NP + M1-RSVF vaccine regimens protected mice against RSV challenge, however, IM-IN- and IN-IN-vaccinated mice had the lowest viral loads upon challenge. The IM-IN regimen, specifically, was more Th1-leaning according to systemic immunogenicity data; IM-IN-vaccinated mice possessed the greatest relative IgG2a levels compared with other subclasses, and the smallest relative IgG1 levels. B cell class-switching to IgG2a is promoted by Th1-associated IFNγ release in the cellular milieu, whereas promotion of class-switching to IgG1 is mediated by Th2-associated cytokine IL-4^60^. In alignment with this, the systemic (spleen) cytokine staining responses observed were generally TNF and IFNγ biased, and IM-IN vaccination was shown to induce high levels of IFNγ as measured via ELISpot assay. Th1 responses have been linked to lessened disease during RSV infection, whereas Th2-type responses have been associated with enhanced disease^42,61,62^.

NAbs targeting the F protein likely contributed a considerable degree to the protection observed against RSV disease^63–65^. Earlier serum and antibody passive transfer studies and more recent correlate of protection studies have supported the role of humoral immunity in protection from RSV infection and disease^63–65^. A large number of RSV candidates, as well as licensed RSV vaccines Abrysvo and Arexvy, utilise stabilised F protein antigens, which have been widely tested and proven to generate protective neutralising antibodies^14,19,66–68^. The lower measured viral load in IM-IN- and IN-IN-vaccinated mouse lungs may have been as a result of enhanced mucosal antibody responses, or may reflect a more multifactorial mechanism of RSV protection. CD8^+^ and CD4^+^ T cells, and specifically T_RM_, have also been described as important for protection against RSV, and in other studies, control over RSV infection has been demonstrated by T cells in the absence of RSV-specific antibodies^69–72^.

Limited work has explored the employment of RSV and influenza vaccines via a heterologous systemic prime, mucosal boost regimen. However, a range of studies involving RSV and influenza vaccines have tested mucosal vaccinations^6,14,15,73–81^. In such examples, mucosal vaccination (via IN, intrapulmonary and/or aerosol) priming generally generated higher respiratory mucosal responses than parenteral/systemic routes (IM and intraperitoneal). Nevertheless, within our study we found that responses of the greatest magnitude and protection against disease and infection were established when systemic priming preceded a mucosal boost; such responses were generally higher in IM-IN-vaccinated mice than IN-prime or IN-IN-vaccinated mice, highlighting the benefit of hybrid, inter-compartmental vaccination. With such a combination, the benefits of systemic and respiratory mucosal immunity are both harnessed. IM-priming “secured” an IgG2a > IgG1 response that was maintained following IN boost.

Alternative systemic-prime, mucosal-boost vaccination regimens have been described in other studies involving SARS-CoV-2 vaccines. One such study used a ChAd-based platform, and showed that a combination IM-IN regimen was able to induce potent responses superior to homologous route regimens, with antibody responses of the highest magnitude and Th1-skewing, consistent with our results^82–85^. The “prime-pull” regimen strategy has been used for other mucosal vaccines against non-respiratory pathogens, such as chlamydia; and proven to induce strong immunity within minipigs^86^.

Further investigation of the suitability of the ChAdOx1-NP + M1-RSVF and the IM-IN vaccination strategy for use in infants and the elderly will be important, as infant and older adults cohorts are particularly burdened by RSV and influenza virus^23,87^. Consideration of current clinical practice regarding vaccination against RSV and influenza and how this vaccine could be used as an auxiliary product will be considered through future clinical development and assessment.

This study supports further exploration of the systemic prime, mucosal boost route regimen combination for vaccines against respiratory viruses. It specifically demonstrates ChAdOx1-NP + M1-RSVF as a bivalent RSV and influenza vaccine candidate suitable for further clinical testing.

## Methods

### Vaccine construction

The nucleoprotein (NP) gene and Matrix protein 1 (M1) gene from influenza virus A/Panama/2007/99 (H3N2) were linked through a glycine linker and codon optimised for expression in human cells. The genes were synthesised by GeneArt Gene Synthesis (Thermo Fisher Scientific). The genes were inserted into the Gateway® attL1/2 recombination cassette of a shuttle plasmid containing a human cytomegalovirus major immediate early promoter (IE CMV), including two tetracycline operator 2 sites, and the bovine growth hormone polyadenylation signal. The separate pre-fusion-stabilised RSV-F (DS2) gene sequence was codon-optimized for expression in human cell lines and synthesised by GeneArt Gene Synthesis (Thermo Fisher Scientific). This gene was inserted into the Gateway® attL3/4 recombination cassette of a shuttle plasmid containing a human cytomegalovirus major immediate early promoter (IE CMV), also including two tetracycline operator 2 sites, and the SV40 polyadenylation signal. Bacterial artificial chromosomes (BACs) containing ChAdOx1 NP + M1-RSVF were prepared by Gateway® recombination between the ChAdOx1 destination DNA BAC vector (described in Dicks, M. D. J. et al.^88^) and the shuttle plasmids containing the influenza virus NP + M1 gene expression cassette and RSVF gene expression cassette using standard protocols resulting in the insertion of the influenza virus gene expression cassette at the E1 locus and RSV gene expression cassette at the E4 locus (downstream of the E4 genes and upstream of the right inverted terminal repeat). The ChAdOx1 NP + M1-RSVF adenovirus genome was excised from the BAC using unique PmeI sites flanking the adenovirus genome sequence. ChAdOx1 NP + M1-RSVF viral vectors were rescued in T-REx^TM^ cells (Invitrogen, Cat. R71007), a derivative of HEK293 cells which constitutively express the Tet repressor protein and prevent antigen expression during virus production. The resultant virus, ChAdOx1 NP + M1-RSVF, was purified by CsCl gradient ultracentrifugation as described previously^89^. The titres were determined on T-RExTM cells using antihexon immunostaining assay based on the QuickTiter™ Adenovirus Titer Immunoassay kit (Cell Biolabs Inc).

### Mouse immunisations and infection studies

Mice for immunogenicity studies were used in accordance with the UK Animals (Scientific Procedures) Act under project license number 30/2889 or P9804B4F1 granted by the UK Home Office following an ethical review by the University of Oxford Animal Welfare and Ethical Review Board (AWERB). Mice for infection studies were used under license PP5168779, with all work approved by the Animal Welfare and Ethical Review board at Imperial College London; studies were in accordance with the Animal Research: Reporting of in vivo Experiments (ARRIVE) guidelines. Animals were group housed in IVCs under SPF conditions, with constant temperature and humidity with lighting on a 12:12 (8am to 8 pm) or 13:11 (7am to 8 pm) light-dark cycle. For induction of short-term anaesthesia, animals were anaesthetised using vaporised IsoFlo^®^. All animals were humanely sacrificed at the end of each experiment by cervical dislocation for immunogenicity studies, or via administration of 100 μL of intraperitoneal pentobarbitone (20 mg dose, Pentoject, Animalcare Ltd., UK) for infection studies. For assessment of vaccine immunogenicity, 5-week-old outbred CD-1 (Envigo) (*n* = 6) mice were vaccinated in regimens containing intramuscular injection and/or intranasal administration with 10^8^ infection units (IU) of vector vaccine diluted in PBS under isofluorane-induced anaesthesia. Intranasal vaccination involved suspending a total volume of 25 μL of vaccine drop-by-drop over the mouse nostrils with a pipette, ensuring active inhalation of the vaccine. Intramuscular vaccinations involved injection of 50 μL of vaccine into the muscle of the left inner thigh. For infection challenge experiments, female BALB/c mice (Charles River, Polcreek) (*n* = 5) were anaesthetised using vaporised IsoFlo^®^ and intranasally infected with 100 µl volume containing virus: RSV - 7.7×10^5^ PFU RSV A2; H1N1 - 2.1×10^5^ PFU A/California/7/2009; H3N2 2×10^5^ X31. Viruses were propagated in cells (RSV:HEp2; Influenza:MDCK); RSV A2 was 3^rd^ passage from a plaque-purified virus isolated by Dr Hongwei Wang (Imperial)^90^; Influenza viruses were a gift from Wendy Barclay (Imperial); they were also low passage. Mice were weighed daily following infection.

### Blood, lung and spleen processing

Blood was collected either by terminal cardiac puncture (immunogenicity studies) or femoral vein (infection studies) and processed via centrifugation to collect serum fraction. Lungs were removed after dissection with the right lobe processed for flow cytometry and the left lobe frozen for future RNA extraction (for infection studies) or both lobes immediately processed for flow cytometry (for immunogenicity studies). For flow cytometry and ELISpots, spleens and lung were processed to a single cell suspension, and reconstituted in complete Minimum Essential Medium, α modification (α-MEM) (for immunogenicity studies), or complete RPMI (for infection studies), containing 10% fetal calf serum (FCS), 1% penicillin/streptomycin and 1% L-glutamine. Lung tissue was treated with a digestion mixture of collagenase type XI and DNAse type I in α-MEM solely containing 1% penicillin/streptomycin and 1% L-glutamine, prior to processing in immunogenicity studies only. Processing of spleens and lungs was completed mechanically by pressing the tissue through 70-100 μM cell strainers, and the use of ammonium chloride potassium (ACK) lysis buffer for erythrocyte lysis. In immunogenicity studies, lung homogenate supernatant (LHS) was collected following the straining and centrifugation of lung cells (prior to their lysis), and was kept frozen until analysis via ELISA. Processed cells were then either manually counted via trypan blue (for infection studies), or via CASY cell counter (for immunogenicity studies).

### Naso-associated lymphoid tissue (NALT) removal and processing

To acquire NALT fluid, the upper hard palate of mice was excised with a scalpel and cleaned of blood and debris. It was then added to 250 μL complete RPMI in wells of a 48 well plate. It was incubated for 3 days at 37 °C in a cell culture incubator. After incubation, supernatant was removed and spun down to pellet cellular debris. Supernatant was then frozen until use in assays. A more detailed protocol is described by Cisney et al.^91^.

### Bronchoalveolar lavage fluid (BALF) collection and nasal washes (NWs)

For BALF collection, a catheter was inserted into the trachea and mouse lungs were then lavaged with a syringe to inflate the lung three times with 1 ml of PBS (for infection studies) or lavaged solely once with 300 µL (for immunogenicity studies). BAL fluid was then transferred to a 1.5 mL collection tube and centrifuged at 460 g for 5 minutes and supernatants collected and frozen at −80 °C (for both infection and immunogenicity studies). Supernatants were additionally treated with protease inhibitor at 1:100 dilution prior to freezing for immunogenicity studies. The cell pellet was then resuspended with 500 µL of ACK for 2 minutes, then quenched with PBS, before centrifugation at 460 g for 5 minutes. The pellet was then resuspended in RPMI for flow cytometry in infection studies. Nasal fluid for NWs were collected by flushing the nasal cavity with 1 mL of PBS.

### Lung cell and splenocyte stimulation, staining and flow cytometry following immunogenicity studies

Each lung and splenocyte sample were stimulated separately with a pool of peptides spanning H3N2 A/Panama/2007/1999 NP + M1 or mRSV(F)-DS2, or were left unstimulated, for 6 hours at 37 °C (Supplementary Table 4a). 2 hours into the incubation, BD Golgiplug^TM^ was added to all cells. After stimulation, cells were stained with surface markers, fixed with 10% neutral-buffered formalin (containing 4% paraformaldehyde), and then permeabilised with BD PERM/Wash^TM^ buffer to allow intracellular antibody staining. Cells were stained with BV711™ anti-mouse CD69 Antibody (Clone: H1.2F3, Cat: 104537 (Biolegend), diluted 1:100), Alexa Fluor® 488 anti-mouse TNF-α Antibody (Clone: MP6-XT22, Cat: 506313 (Biolegend), diluted 1:100), PE anti-mouse IL-4 Antibody (Clone: 11B11, Cat: 504104 (Biolegend), dilution 1:50), PE/Dazzle™ 594 anti-mouse CD103 Recombinant Antibody (Clone: QA17A24, Cat: 156910 (Biolegend), diluted 1:50), PE/Cyanine7 anti-mouse CD62L Antibody (Clone: MEL-14, Cat: 104418 (Biolegend), diluted 1:50), Alexa Fluor® 700 anti-mouse/human CD44 Antibody (Clone: IM7, Cat: 103026 (eBioscience), diluted 1:100), BV650™ anti-mouse/human CD45R/B220 Antibody (Clone: RA3-62B, Cat: 103241 (Biolegend), diluted 1:100), IFN gamma Monoclonal Antibody eFluor 450 (Clone: XMG1.2, Cat: 48-7311-82 (eBioscience), diluted 1:50), BUV496 Rat Anti-Mouse CD4 (Clone: GK1.5, Cat: 612952 (BD Biosciences), diluted 1:100), BUV395 Rat Anti-Mouse CD8a (Clone: 53-6.7, Cat: 563786 (BD Biosciences), diluted 1:100), PerCP/Cyanine5.5 anti-mouse IL-2 Antibody (Clone: JES6-5H4, Cat: 503822 (Biolegend), diluted 1:50), PE/Cyanine5 anti-mouse CD127 (IL-7Rα) Antibody (Clone: A7R34, Cat: 135016 (Biolegend), diluted 1:100) and BV605™ anti-mouse CD183 (CXCR3) Antibody (Clone: CXCR3-173, Cat: 126523 (Biolegend), diluted 1:100). Excess antibodies were washed off with 1% BSA in PBS three times then acquired on a BD LSRFortessa™ Cell Analyzer. To stain for T_RM_ cells, the panel above was modified to include Brilliant Violet 650^TM^ anti-mouse CD3 (Clone: 172 A, Cat: 100229 (Biolegend)) that was intravenously injected into the peripheral tail vein of mice before culling (2.5 μg per mouse in 100 μL total injection volume). BV650™ anti-mouse/human CD45R/B220 antibody was not included in this alternative panel.

### Cell staining and flow cytometry following infection studies

Lung cells and cells from BALF were first stained with 100 µl of Live/Dead violet dye (ArCTM, Cat: A10346) for 20 minutes at 4 °C in the dark. After washing, cells were then resuspended in Fc block (Clone: 2.4G2) in PBS-1% BSA, then washed and stained with the following surface antibodies: FITC anti-mouse CD3 (Clone: 12A2, Cat: 100204 (Biolegend), diluted 1:400), APC-H7 anti-CD8 (Clone: 53-6.7, Cat: 560247 (BD Biosciences), diluted 1:100), BV605 anti-CD103 (Clone: 2E7, Cat: 121433 (Biolegend), diluted 1:70), APC anti-mouse CD69 (Clone: H1.2F3, Cat: 104513 (Biolegend), diluted 1:70), PerCP-Cy5.5 anti-mouse CD4 (Clone: RM4-5, Cat: 100540 (Biolegend), diluted 1:200), BV711 anti-mouse CD44 (Clone: IM7, Cat: 103001 (Biolegend), diluted 1:200), PE-Cy7 anti-mouse CD62L (Clone: MEL-14, Cat: 104418 (Biolegend), diluted 1:200) for one hour in the dark. For influenza infection experiments, pentamer H-2Kd TYQRTRALV (Influenza A (PR8) NP 147-155: ProImmune, diluted 1:100) was additionally added to the surface antibody stain. For RSV infection experiment, pentamer H-2Kd KYKNVTEL (RSV A F protein 85-93: ProImmune, diluted 1:100) was additionally added to the surface antibody stain; pentamers were PE conjugated. Excess antibodies were washed off with 1% BSA in PBS three times then acquired on a BD LSRFortessa™ Cell Analyzer.

### Spleen IFNγ ELISpots

PVDF-membrane ELISpot plates (Millipore) were coated with 5μg/mL anti-mouse IFNy (Cat: 3321-2 A, AN18 (Mabtech)), 50 μL per well. Splenocytes in complete α-MEM, at a density of 10^7^ cells/mL, were plated on pre-blocked plates. Then, peptide pools either spanning NP + M1 or RSVF-DS2 (4 total pools per antigen) were added for stimulation of the cells over 18 hours at 37^ o^C (Supplementary Table 4a). IFNy spots were detected through the use of anti-mouse IFNγ antibody, biotinylated (Cat: 3321-2 A, mAb R4-6A2, biotin (Mabtech), diluted 1 mg/mL), followed by streptavidin-ALP (Cat: 3321-2 A (Mabtech), diluted 1 mg/mL). Development involved adding AP conjugate substrate (Cat: 1706432 (Biorad)). Spots were counted on an AID ELISpot reader, and data represented as spot-forming units (SFUs) per million splenocytes. The sum of responses from one sample across the 4 antigen peptide pools was used for figures.

### Lung viral load measurement

Viral load was assessed by Trizol extraction of RNA from frozen lung tissue disrupted in a TissueLyzer (Qiagen, Manchester, UK). RNA was converted into cDNA, and quantitative RT-PCR was carried out on a Stratagene Mx3005p (Agilent Technologies, Santa Clara, CA, USA). For influenza M gene 0.1 μM forward primer (5′AAGACAAGACCAATYCTGTCACCTCT-3′), 0.1 μM reverse primer (5′-TCTACGYTGCAGTCCYCGCT-3′) and 0.2 μM probe (5′-FAM-TYACGCTCACCGTGCCCAGTG-TAMRA-3′). M-specific RNA copy number was determined using an influenza M gene standard plasmid. For RSV L gene, 0.1 µM primers 5′-GAACTCAGTGTAGGTAGAATGTTTGCA-3′ and 5′-TTCAGCTATCATTTTCTCTGCCAA-3′ and probe 5′-6-carboxyfluorescein (FAM)-TTTGAACCTGTCTGAACAT-6-carboxytetramethylrhodamine (TAMRA)-3′. RNA copy number per mg of lung RNA was determined using an RSV L gene standard.

### Standardised indirect antigen-specific isotype ELISAs

96 well Nunc^TM^MaxiSorp^TM^ plates were separately coated with 50μL/well of 2μg/mL (for tIgG and IgG subclass detection) or 5μg/mL (for IgA and IgM detection) recombinant Influenza A H1N1 (A/Puerto Rico/8/34/Mount Sinai) Nucleoprotein/NP I116M (ECD, His Tag), Influenza A H1N1 (A/Puerto Rico/8/34/Mount Sinai) Matrix protein 1 / M1 Protein (His Tag), Influenza A H3N2 (A/Hong Kong/2671/2019) Nucleoprotein / NP Protein (His Tag) Influenza A H3N2 (A/Aichi/2/1968) Matrix protein 1 / M1 Protein (His Tag) or Human respiratory syncytial virus (RSV) (A2) Fusion glycoprotein/RSV-F protein (ECD, His Tag) (all SinoBiological), overnight at 4 °C. Plates were then washed with PBS/Tween (0.05% v/v), and then blocked for 1 hour at RT with Blocker Casein in PBS (Thermo Fisher Scientific). Blocker was then tapped off, and standard positive “high-responding” serum or mucosal tissue fluid was added, along with samples at individual dilutions in casein, and other appropriate controls, all in duplicate; sample incubation was 2 hours at RT for all assays. Plates were washed after incubation, and casein-diluted secondary antibody added to plates for incubation 1 hour RT shaking; antibodies used were alkaline phosphatase (AP)-conjugated goat anti-mouse IgG (Cat:A3562 (Sigma Aldrich), diluted 1:5000), Goat Anti-Mouse IgA-AP (Cat: 1040-04 (SouthernBiotech), diluted 1:1500), Goat Anti-Mouse IgM-AP (Cat: 1021-04 (SouthernBiotech), diluted 1:1500), Goat Anti-Mouse IgG1, Human/Bovine/Horse SP ads-AP (Cat: 1071-04 (SouthernBiotech), diluted 1:4000), Goat Anti-Mouse IgG2a, Human/Bovine/Horse SP ads-AP (Cat: 1081-04 (SouthernBiotech), diluted 1:4000), Goat Anti-Mouse IgG2b, Human/Bovine/Horse SP ads-AP (Cat: 1091-04 (SouthernBiotech), diluted 1:4000), Goat Anti-Mouse IgG2c, Human/Bovine/Horse SP ads-AP (Cat: 1078-04 (SouthernBiotech), diluted 1:4000), Goat Anti-Mouse IgG3 heavy chain (Alkaline Phosphatase) preadsorbed (Cat: ab98705 (Abcam), diluted 1:1000). Unbound detection antibody was washed prior to addition of p-Nitrophenyl Phosphate, Disodium Salt substrate (Sigma-Aldrich). For IgG, IgA and IgM sample, OD_405nm_ values were interpolated off a standard curve made of positive reference sera, which was assigned arbitrary ELISA units and fitted to a 4-parameter logistic curve. For IgG subclasses, sera samples were normalised based on tIgG values prior to plating, and plates read when the first sample in any group reached 1.00 OD_405nm_ for each subclass.

### Data and statistical analyses

All statistical analyses were performed on GraphPad Prism 9.0 and 10.0. Medians were used as representative values for each mouse group. Solely nonparametric statistical tests were completed on data. For paired data (such as vaccination timepoint antibody data), Friedman tests were used to determine statistically significant differences. For differences between comparable groups, such as different vaccination regimens, Kruskal Wallis tests were completed. For assessment of statistically significant differences in experiments involving two groups, Mann-Whitney U-Tests were performed. *P* values were symbolised as *p < 0.05, **p < 0.01, ***p < 0.001. Where data was represented in log_10_ form, data was converted to log_10_ prior to statistical analysis. Flow data was analysed on FlowJo software (10.9.0) using the gating strategies described in Supplementary Fig. 7a and b. For cytokine expression and ELISpot values displayed on graphs, the values from the samples when they were in an unstimulated state were subtracted from the stimulated values.

### Supplementary information

Supplementary information

## Data Availability

The datasets used and/or analysed during the current study are available from the corresponding author on reasonable request.

## References

[1] Sridhar S, Brokstad KA, Cox RJ (2015). Influenza Vaccination Strategies: Comparing Inactivated and Live Attenuated Influenza Vaccines. Vaccines.

[2] Oh JE (2021). Intranasal priming induces local lung-resident B cell populations that secrete protective mucosal antiviral IgA. Sci. Immunol..

[3] Fu Y-H (2013). Intranasal immunization with a helper-dependent adenoviral vector expressing the codon-optimized fusion glycoprotein of human respiratory syncytial virus elicits protective immunity in BALB/c mice. Virol. J..

[4] Khan IU, Huang J, Li X, Xie J, Zhu N (2018). Nasal immunization with RSV F and G protein fragments conjugated to an M cell-targeting ligand induces an enhanced immune response and protection against RSV infection. Antivir. Res.

[5] Mossad SB (2003). Demystifying FluMist, a new intranasal, live influenza vaccine. Clevel. Clin. J. Med..

[6] Calzas C, Chevalier C (2019). Innovative Mucosal Vaccine Formulations Against Influenza A Virus Infections. Front. Immunol..

[7] Cokarić Brdovčak M (2022). ChAdOx1-S adenoviral vector vaccine applied intranasally elicits superior mucosal immunity compared to the intramuscular route of vaccination. Eur. J. Immunol..

[8] Tang J (2022). Respiratory mucosal immunity against SARS-CoV-2 after mRNA vaccination. Sci. Immunol..

[9] Hameed SA, Paul S, Dellosa GKY, Jaraquemada D, Bello MB (2022). Towards the future exploration of mucosal mRNA vaccines against emerging viral diseases; lessons from existing next-generation mucosal vaccine strategies. npj Vaccin..

[10] Puksuriwong S (2019). MVA-NP+M1 vaccine activates mucosal M1-specific T cell immunity and tissue-resident memory T cells in human nasopharynx-associated lymphoid tissue. J. Infect. Dis..

[11] Morgan SB (2016). Aerosol Delivery of a Candidate Universal Influenza Vaccine Reduces Viral Load in Pigs Challenged with Pandemic H1N1 Virus. J. Immunol..

[12] XS H (2006). Cellular immune responses in children and adults receiving inactivated or live attenuated influenza vaccines. J. Virol..

[13] Mohn KGI, Zhou F, Brokstad KA, Sridhar S, Cox RJ (2017). Boosting of Cross-Reactive and Protection-Associated T Cells in Children After Live Attenuated Influenza Vaccination. J. Infect. Dis..

[14] Vlachantoni I (2017). S68 Phase 1 trial of an intranasal respiratory syncytial virus (rsv) subunit candidate vaccine: safety results from the muc-syngem study. Thorax.

[15] Ivanov V (2021). Intranasal and intrapulmonary vaccination with an M protein-deficient respiratory syncytial virus (RSV) vaccine improves clinical signs and reduces viral replication in infant baboons after an RSV challenge infection. Vaccine.

[16] Study Record | Beta ClinicalTrials.gov. https://clinicaltrials.gov/study/NCT05655182.

[17] Shan J, Britton PN, King CL, Booy R (2021). The immunogenicity and safety of respiratory syncytial virus vaccines in development: A systematic review. Influenza Other Respirat. Viruses.

[18] Soto JA (2020). Current Insights in the Development of Efficacious Vaccines Against RSV. Front. Immunol..

[19] McLellan JS (2013). Structure-Based Design of a Fusion Glycoprotein Vaccine for Respiratory Syncytial Virus. Sci. (N. Y., N. Y.).

[20] Swanson KA (2011). Structural basis for immunization with postfusion respiratory syncytial virus fusion F glycoprotein (RSV F) to elicit high neutralizing antibody titers. Proc. Natl Acad. Sci. USA.

[21] McLellan JS, Yang Y, Graham BS, Kwong PD (2011). Structure of Respiratory Syncytial Virus Fusion Glycoprotein in the Postfusion Conformation Reveals Preservation of Neutralizing Epitopes. J. Virol..

[22] Krarup A (2015). A highly stable prefusion RSV F vaccine derived from structural analysis of the fusion mechanism. Nat. Commun..

[23] Respiratory syncytial virus (RSV) immunisation programme for infants and older adults: JCVI full statement, 11 September 2023. *GOV.UK* https://www.gov.uk/government/publications/rsv-immunisation-programme-jcvi-advice-7-june-2023/respiratory-syncytial-virus-rsv-immunisation-programme-for-infants-and-older-adults-jcvi-full-statement-11-september-2023.

[24] Krammer, F. The human antibody response to influenza A virus infection and vaccination. 10.1038/s41577-019-0143-6. 10.1038/s41577-019-0143-6 30837674

[25] Rajão DS, Pérez DR (2018). Universal vaccines and vaccine platforms to protect against influenza viruses in humans and agriculture. Front. Microbiol..

[26] Antrobus RD (2014). Clinical Assessment of a Novel Recombinant Simian Adenovirus ChAdOx1 as a Vectored Vaccine Expressing Conserved Influenza A Antigens. Mol. Ther..

[27] McMahon M (2019). Vaccination with viral vectors expressing chimeric hemagglutinin, NP and M1 antigens protects ferrets against influenza virus challenge. Front. Immunol..

[28] Saunders JE (2023). Adenoviral vectored vaccination protects against Crimean-Congo Haemorrhagic Fever disease in a lethal challenge model. eBioMedicine.

[29] Voysey M (2021). Safety and efficacy of the ChAdOx1 nCoV-19 vaccine (AZD1222) against SARS-CoV-2: an interim analysis of four randomised controlled trials in Brazil, South Africa, and the UK. Lancet.

[30] Bosaeed M (2022). Safety and immunogenicity of ChAdOx1 MERS vaccine candidate in healthy Middle Eastern adults (MERS002): an open-label, non-randomised, dose-escalation, phase 1b trial. Lancet Microbe.

[31] Jenkin D (2023). Safety and immunogenicity of a ChAdOx1 vaccine against Rift Valley fever in UK adults: an open-label, non-randomised, first-in-human phase 1 clinical trial. Lancet Infect. Dis..

[32] van Doremalen N (2022). ChAdOx1 NiV vaccination protects against lethal Nipah Bangladesh virus infection in African green monkeys. npj Vaccin..

[33] van Doremalen N (2021). Intranasal ChAdOx1 nCoV-19/AZD1222 vaccination reduces viral shedding after SARS-CoV-2 D614G challenge in preclinical models. Sci. Transl. Med.

[34] Van Erp EA, Luytjes W, Ferwerda G, Van Kasteren PB (2019). Fc-mediated antibody effector functions during respiratory syncytial virus infection and disease. Front. Immunol..

[35] Thwaites RS (2023). Early mucosal events promote distinct mucosal and systemic antibody responses to live attenuated influenza vaccine. Nat. Commun..

[36] Baker JR, Farazuddin M, Wong PT, O’Konek JJ (2022). The unfulfilled potential of mucosal immunization. J. Allergy Clin. Immunol..

[37] Iwasaki, A. Exploiting Mucosal Immunity for Antiviral Vaccines. (2016) 10.1146/annurev-immunol-032414-112315. 10.1146/annurev-immunol-032414-112315 27168245

[38] Pizzolla A (2017). Resident memory CD8+ T cells in the upper respiratory tract prevent pulmonary influenza virus infection. Sci. Immunol..

[39] Tang J, Sun J (2023). Lung tissue-resident memory T cells: the gatekeeper to respiratory viral (re)-infection. Curr. Opin. Immunol..

[40] Zens KD, Chen JK, Farber DL (2016). Vaccine-generated lung tissue–resident memory T cells provide heterosubtypic protection to influenza infection. JCI Insight.

[41] Taylor G (2017). Animal models of respiratory syncytial virus infection. Vaccine.

[42] Waris ME, Tsou C, Erdman DD, Zaki SR, Anderson LJ (1996). Respiratory synctial virus infection in BALB/c mice previously immunized with formalin-inactivated virus induces enhanced pulmonary inflammatory response with a predominant Th2-like cytokine pattern. J. Virol..

[43] Mytle N (2021). Influenza Antigens NP and M2 Confer Cross Protection to BALB/c Mice against Lethal Challenge with H1N1, Pandemic H1N1 or H5N1 Influenza A Viruses. Viruses.

[44] Lin H-T, Chuang C-C, Wu H-L, Chu D-M, Wang Y-C (2013). Characterization of cross protection of Swine-Origin Influenza Virus (S-OIV) H1N1 and reassortant H5N1 influenza vaccine in BALB/c mice given a single-dose vaccination. J. Biomed. Sci..

[45] Balazs AB, Bloom JD, Hong CM, Rao DS, Baltimore D (2013). Broad protection against influenza infection by vectored immunoprophylaxis in mice. Nat. Biotechnol..

[46] Rappazzo CG (2016). Recombinant M2e outer membrane vesicle vaccines protect against lethal influenza A challenge in BALB/c mice. Vaccine.

[47] Lee S (2012). Vaccine-Elicited CD8+ T Cells Protect against Respiratory Syncytial Virus Strain A2-Line19F-Induced Pathogenesis in BALB/c Mice. J. Virol..

[48] Roe MK (2022). An RSV Live-Attenuated Vaccine Candidate Lacking G Protein Mucin Domains Is Attenuated, Immunogenic, and Effective in Preventing RSV in BALB/c Mice. J. Infect. Dis..

[49] Stokes KL (2011). Differential pathogenesis of respiratory syncytial virus clinical isolates in BALB/c mice. J. Virol..

[50] Li Y, Li Z, Zhao Y, Chen X (2021). Potentiation of Recombinant NP and M1-Induced Cellular Immune Responses and Protection by Physical Radiofrequency Adjuvant. Vaccines (Basel).

[51] Vatzia E (2021). Respiratory and Intramuscular Immunization With ChAdOx2-NPM1-NA Induces Distinct Immune Responses in H1N1pdm09 Pre-Exposed Pigs. Front Immunol..

[52] Rowell J (2019). The effect of respiratory viruses on immunogenicity and protection induced by a candidate universal influenza vaccine in mice. PLoS One.

[53] Evans TG (2022). Efficacy and safety of a universal influenza A vaccine (MVA-NP+M1) in adults when given after seasonal quadrivalent influenza vaccine immunisation (FLU009): a phase 2b, randomised, double-blind trial. Lancet Infect. Dis..

[54] Vanderven HA (2016). What Lies Beneath: Antibody Dependent Natural Killer Cell Activation by Antibodies to Internal Influenza Virus Proteins. EBioMedicine.

[55] Rijnink WF (2023). Characterization of non-neutralizing human monoclonal antibodies that target the M1 and NP of influenza A viruses. J. Virol..

[56] LaMere MW (2011). Contributions of Antinucleoprotein IgG to Heterosubtypic Immunity against Influenza Virus. J. Immunol..

[57] Fujimoto Y (2016). Cross-protective potential of anti-nucleoprotein human monoclonal antibodies against lethal influenza A virus infection. J. Gen. Virol..

[58] Vanderven HA (2022). Poor protective potential of influenza nucleoprotein antibodies despite wide prevalence. Immunol. Cell Biol..

[59] Groves HT (2018). Mouse Models of Influenza Infection with Circulating Strains to Test Seasonal Vaccine Efficacy. Front Immunol..

[60] Collins AM (2016). IgG subclass co-expression brings harmony to the quartet model of murine IgG function. Immunol. Cell Biol..

[61] Geevarghese B, Weinberg A (2014). Cell-mediated immune responses to respiratory syncytial virus infection. Hum. Vaccin Immunother..

[62] Acosta PL, Caballero MT, Polack FP (2016). Brief History and Characterization of Enhanced Respiratory Syncytial Virus Disease. Clin. Vaccin. Immunol..

[63] Prince GA, Horswood RL, Camargo E, Koenig D, Chanock RM (1983). Mechanisms of immunity to respiratory syncytial virus in cotton rats. Infect. Immun..

[64] Walsh EE, Schlesinger JJ, Brandriss MW (1984). Protection from respiratory syncytial virus infection in cotton rats by passive transfer of monoclonal antibodies. Infect. Immun..

[65] Fong Y (2023). Antibody Correlates of Protection From Severe Respiratory Syncytial Virus Disease in a Vaccine Efficacy Trial. Open Forum Infect. Dis..

[66] Joyce MG (2016). Iterative structure-based improvement of a fusion-glycoprotein vaccine against RSV. Nat. Struct. Mol. Biol..

[67] Mclellan, J. S. et al. Structure of RSV Fusion Glycoprotein Trimer Bound to a Prefusion-Specific Neutralizing Antibody. 10.1126/science.1234914. 10.1126/science.1234914 PMC4459498 23618766

[68] Zhang B (2017). Protection of calves by a prefusion-stabilized bovine RSV F vaccine. NPJ Vaccines.

[69] Endt K (2022). A Recombinant MVA-Based RSV Vaccine Induces T-Cell and Antibody Responses That Cooperate in the Protection Against RSV Infection. Front. Immunol..

[70] De C (2023). Human T cells efficiently control RSV infection. JCI Insight.

[71] Luangrath MA, Schmidt ME, Hartwig SM, Varga SM (2021). Tissue-Resident Memory T Cells in the Lungs Protect against Acute Respiratory Syncytial Virus Infection. Immunohorizons.

[72] Jozwik A (2015). RSV-specific airway resident memory CD8+ T cells and differential disease severity after experimental human infection. Nat. Commun..

[73] Lindell, D. M. et al. A novel inactivated intranasal respiratory syncytial virus vaccine promotes viral clearance without TH2 associated Vaccine-Enhanced disease. *PLoS ONE* **6**, (2011). 10.1371/journal.pone.0021823 PMC3137595 21789184

[74] Boyce TG (1999). Mucosal immune response to trivalent live attenuated intranasal influenza vaccine in children. Vaccine.

[75] Takamura S (2010). The route of priming influences the ability of respiratory virus-specific memory CD8+ T cells to be activated by residual antigen. J. Exp. Med..

[76] Price, G. E. et al. Single-dose mucosal immunization with a candidate universal influenza vaccine provides rapid protection from virulent H5N1, H3N2 and H1N1 viruses. *PLoS ONE* **5** (2010). 10.1371/journal.pone.0013162 PMC2953831 20976273

[77] Pierantoni A (2015). Mucosal delivery of a vectored RSV vaccine is safe and elicits protective immunity in rodents and nonhuman primates. Mol. Ther. - Methods Clin. Dev..

[78] Li H, Ren H, Zhang Y, Cao L, Xu W (2021). Intranasal vaccination with a recombinant protein CTA1-DD-RBF protects mice against hRSV infection. Sci. Rep..

[79] Etchart N (2006). Intranasal immunisation with inactivated RSV and bacterial adjuvants induces mucosal protection and abrogates eosinophilia upon challenge. Eur. J. Immunol..

[80] Verdijk P (2020). First-in-human administration of a live-attenuated RSV vaccine lacking the G-protein assessing safety, tolerability, shedding and immunogenicity: a randomized controlled trial. Vaccine.

[81] Karron, R. A. et al. Evaluation of the live-attenuated intranasal respiratory syncytial virus (RSV) vaccine RSV/6120/ΔNS2/1030s in RSV-seronegative young children. *J. Infect. Dis.* jiad281 10.1093/infdis/jiad281 (2023). 10.1093/infdis/jiad281 PMC10873187 37493269

[82] Lapuente D (2021). Protective mucosal immunity against SARS-CoV-2 after heterologous systemic prime-mucosal boost immunization. Nat. Commun..

[83] Li X (2022). Combining intramuscular and intranasal homologous prime-boost with a chimpanzee adenovirus-based COVID-19 vaccine elicits potent humoral and cellular immune responses in mice. Emerg. Microbes Infect..

[84] Shamseldin MM (2023). Prime-Pull Immunization of Mice with a BcfA-Adjuvanted Vaccine Elicits Sustained Mucosal Immunity That Prevents SARS-CoV-2 Infection and Pathology. J. Immunol..

[85] Mao T (2022). Unadjuvanted intranasal spike vaccine elicits protective mucosal immunity against sarbecoviruses. Science.

[86] Lorenzen E (2015). Intramuscular Priming and Intranasal Boosting Induce Strong Genital Immunity Through Secretory IgA in Minipigs Infected with Chlamydia trachomatis. Front Immunol..

[87] Sullivan SG, Price OH, Regan AK (2019). Burden, effectiveness and safety of influenza vaccines in elderly, paediatric and pregnant populations. Ther. Adv. Vaccines Immunother..

[88] Dicks MDJ (2012). A Novel Chimpanzee Adenovirus Vector with Low Human Seroprevalence: Improved Systems for Vector Derivation and Comparative Immunogenicity. PLoS One.

[89] Cottingham MG (2012). Preventing Spontaneous Genetic Rearrangements in the Transgene Cassettes of Adenovirus Vectors. Biotechnol. Bioeng..

[90] Wang H, Peters N, Schwarze J (2006). Plasmacytoid dendritic cells limit viral replication, pulmonary inflammation, and airway hyperresponsiveness in respiratory syncytial virus infection. J. Immunol..

[91] Cisney, E. D., Fernandez, S., Hall, S. I., Krietz, G. A. & Ulrich, R. G. Examining the Role of Nasopharyngeal-associated Lymphoreticular Tissue (NALT) in Mouse Responses to Vaccines. *JoVE (Journal of Visualized Experiments)* e3960 10.3791/3960 (2012). 10.3791/3960 PMC3476754 22871688

