# Supplementary material for: Systemic prime mucosal boost significantly increases protective efficacy of bivalent RSV influenza viral vectored vaccine
Source: NPJ Vaccines. 2024 Jun 26;9:118. doi: 10.1038/s41541-024-00912-1 (PMC11208422; doi:10.1038/s41541-024-00912-1)
Supplement: Supplementary file 1 — Supplementary information [file 41541_2024_912_MOESM1_ESM.pdf]

Supplementary Figure 1

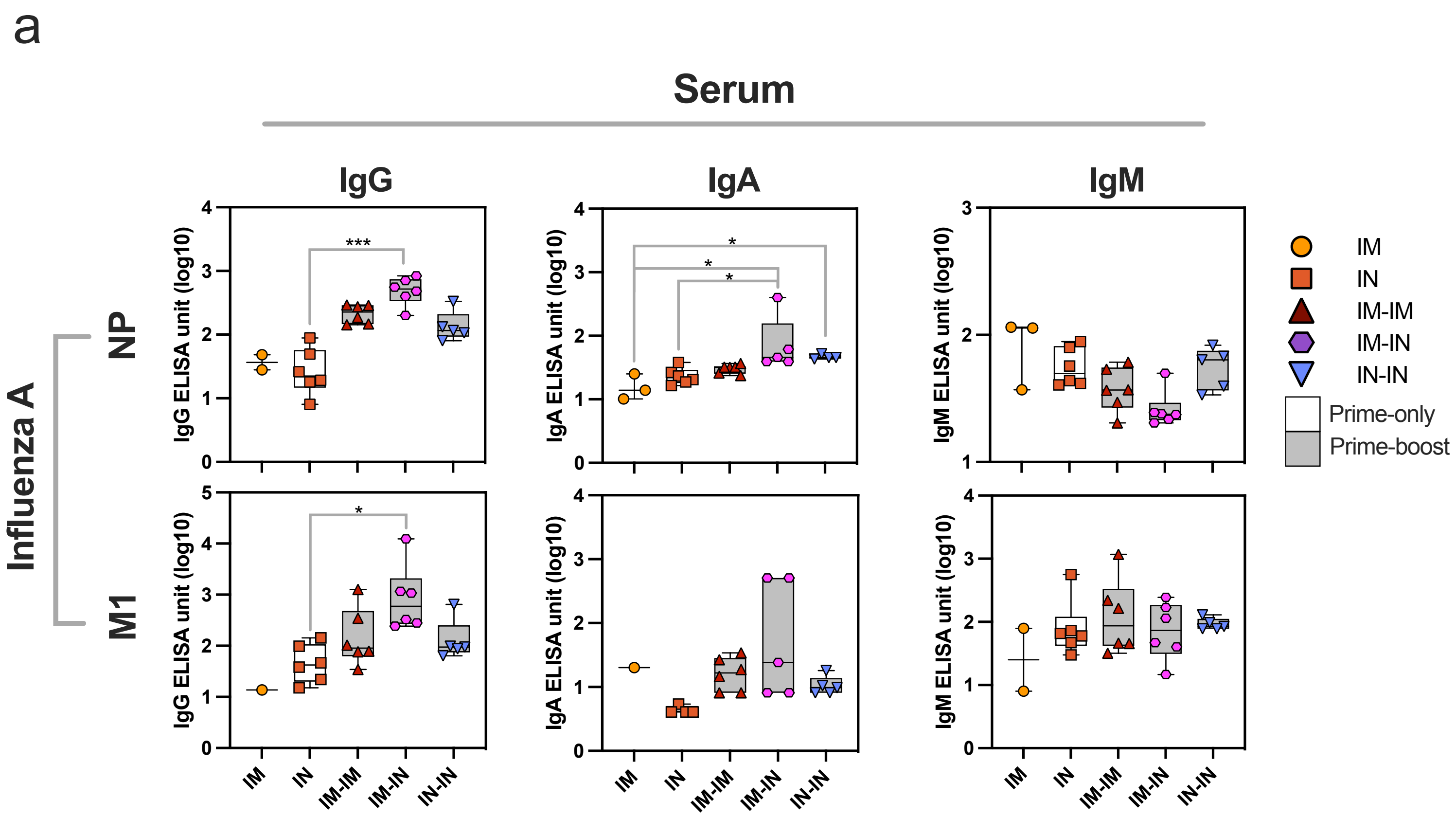

Supplementary Figure 1: (a) IgG, IgA and IgM responses against influenza A H3N2 NP and M1 in sera of mice three weeks post-final vaccination, measured by standardised, indirect ELISA. Values are displayed as EUs in log form. Individual values per mouse are represented as symbols. White bars represent prime-only regimens, and grey shaded bars prime-boost regimens. For all boxplots, whisker endings represent upper and lower extremes, the box bounds represent upper and lower quartiles, respectively, and the central line represents the group median. Values were analysed using nonparametric Kruskal-Wallis tests to assess for statistically significant differences between vaccine regimen groups, which are then expressed in p values (*=p<0.05, **=p<0.01, ***=p<0.001). As such ELISAs were conducted at the end of the study, limited volumes of sample remained. Additionally, multiple freeze-thaw cycles imparted a level of variability to assay results and instability to samples.

Supplementary Figure 2

a

Correlation of anti-NP and anti-M1 IgGA

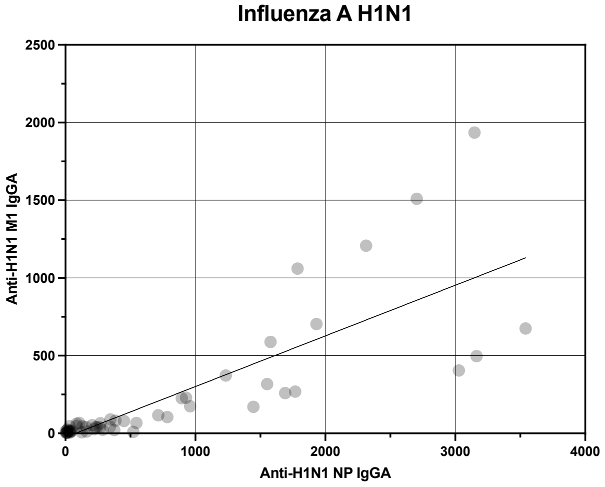

	NP H1N1 vs. M1 H1N1
Spearman r	
r	0.8841
95% confidence interval	0.8065 to 0.9317
P value	
P (two-tailed)	<0.0001
P value summary	****

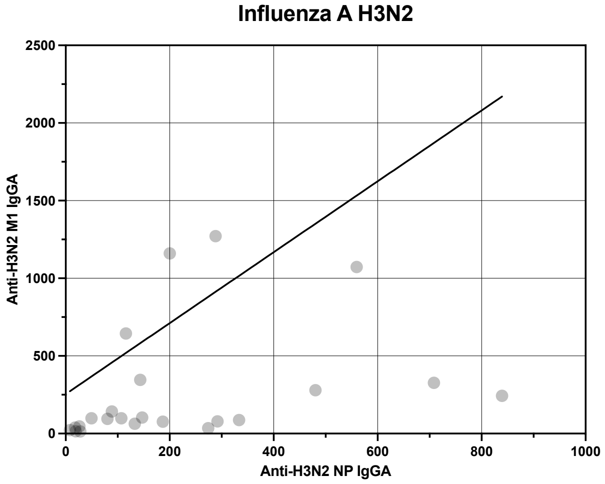

	NP H3N2 vs. M1 H3N2
Spearman r	
r	0.6235
95% confidence interval	0.2824 to 0.8246
P value	
P (two-tailed)	0.0011
P value summary	**

b

Correlation of anti-H3N2 and anti-H1N1 IgGA

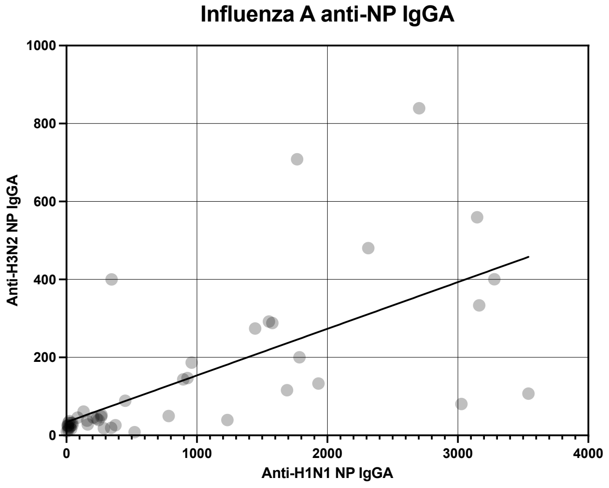

	NP H1N1 vs. H3N2
Spearman r	
r	0.7934
95% confidence interval	0.6526 to 0.8813
P value	
P (two-tailed)	<0.0001
P value summary	****

Supplementary Figure 2: (a) Correlation of anti-H1N1 NP and M1 IgG and IgA (IgGA) and correlation of anti-H3N2 NP and M1 IgGA. Nonparametric Spearman correlations were completed to calculate r values. Simple linear regression lines were plotted on scatter plots. (b) Correlation of anti-H3N2 and anti-H1N1 NP IgGA.

Supplementary Figure 3

a

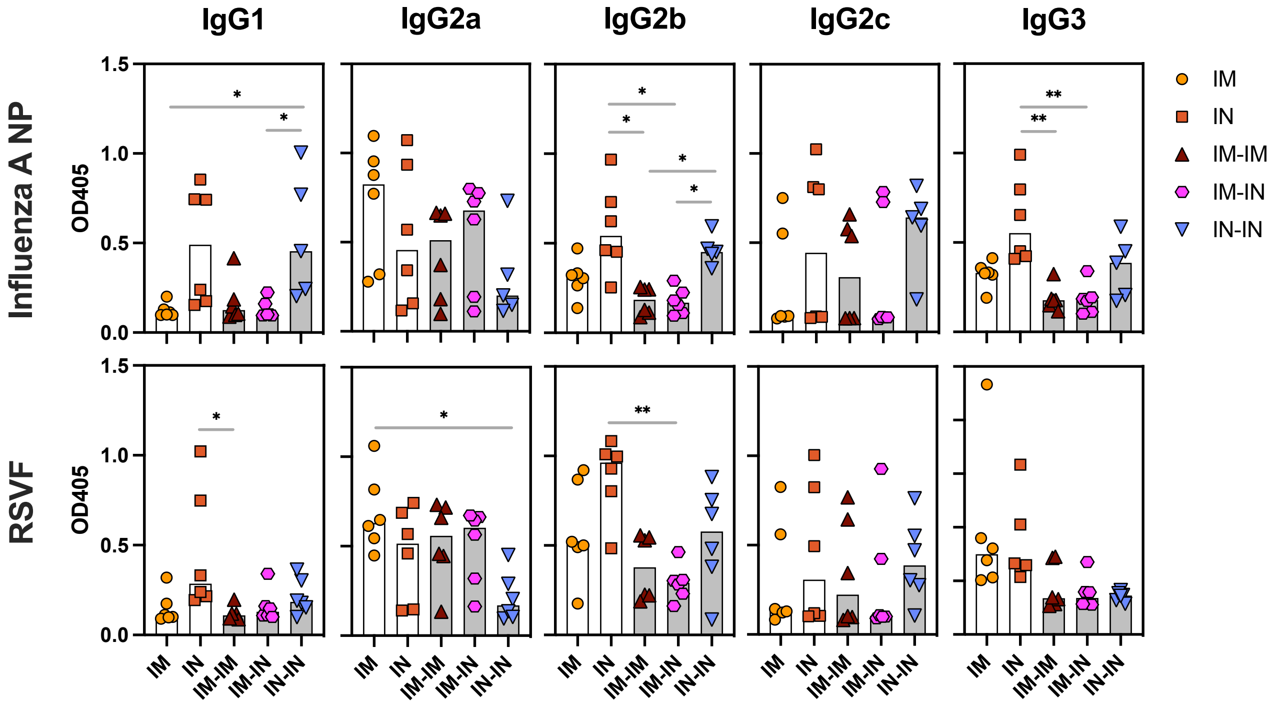

Supplementary Figure 3: (a) Anti-influenza NP and RSVF IgG1, IgG2a, IgG2b, IgG2c and IgG3 in serum of mice following IM, IN, IM-IM, IM-IN, or IN-IN vaccination. Values are demonstrated as OD405s, with the first sample to reach 1.00 OD per subclass per antigen defining the stopping point. The upper bound of each bar represents the group median. Values were analysed using nonparametric Kruskal-Wallis tests to assess for statistically significant differences between vaccine regimen groups, which are then expressed in p values (*=p<0.05, **=p<0.01).

Supplementary Figure 4

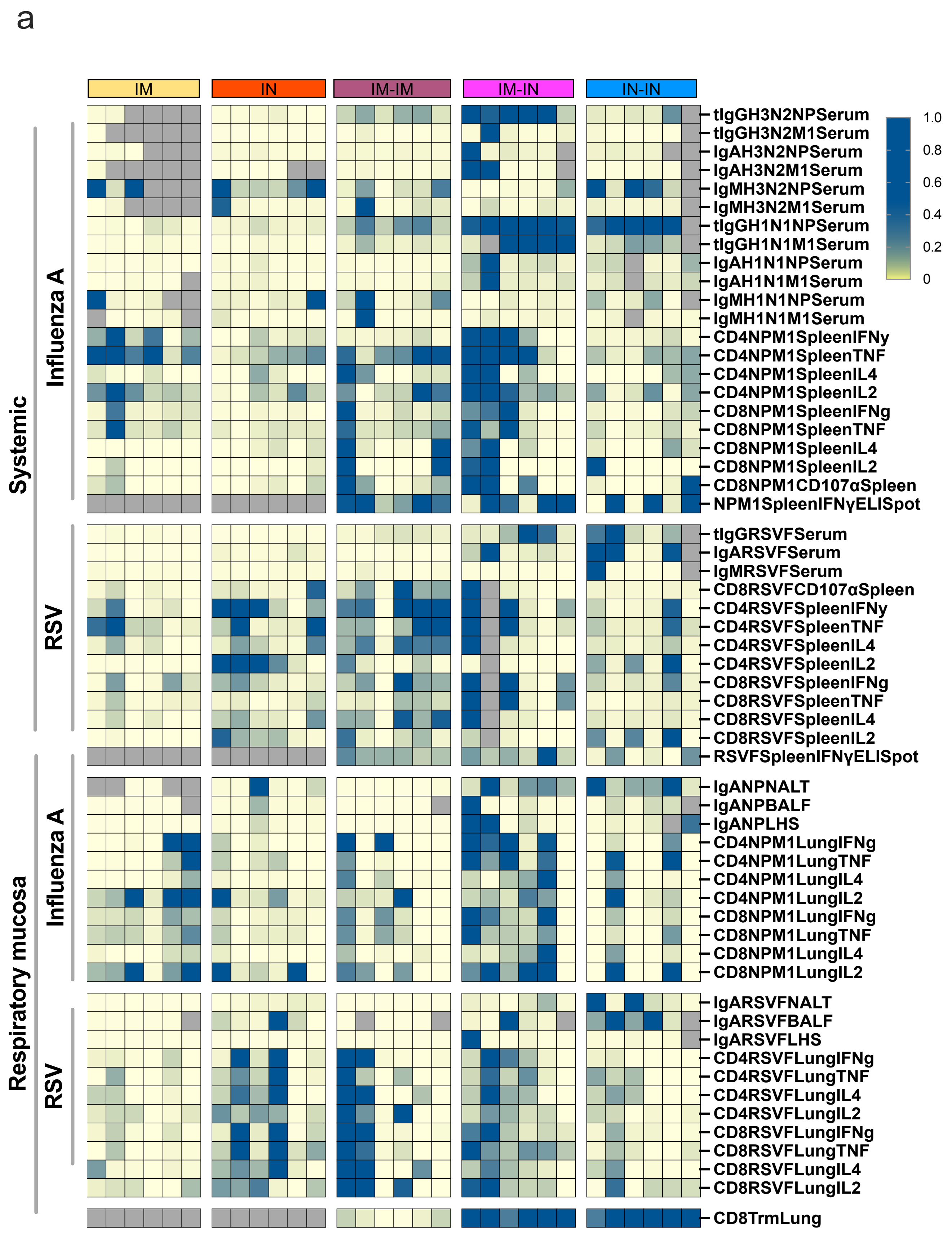

Supplementary Figure 4: (a) Heatmap of compiled immunogenicity responses following different vaccination regimens. Data was min-max (1.0=max, 0.0=min) normalised across regimens per assay readout.

Supplementary Figure 5

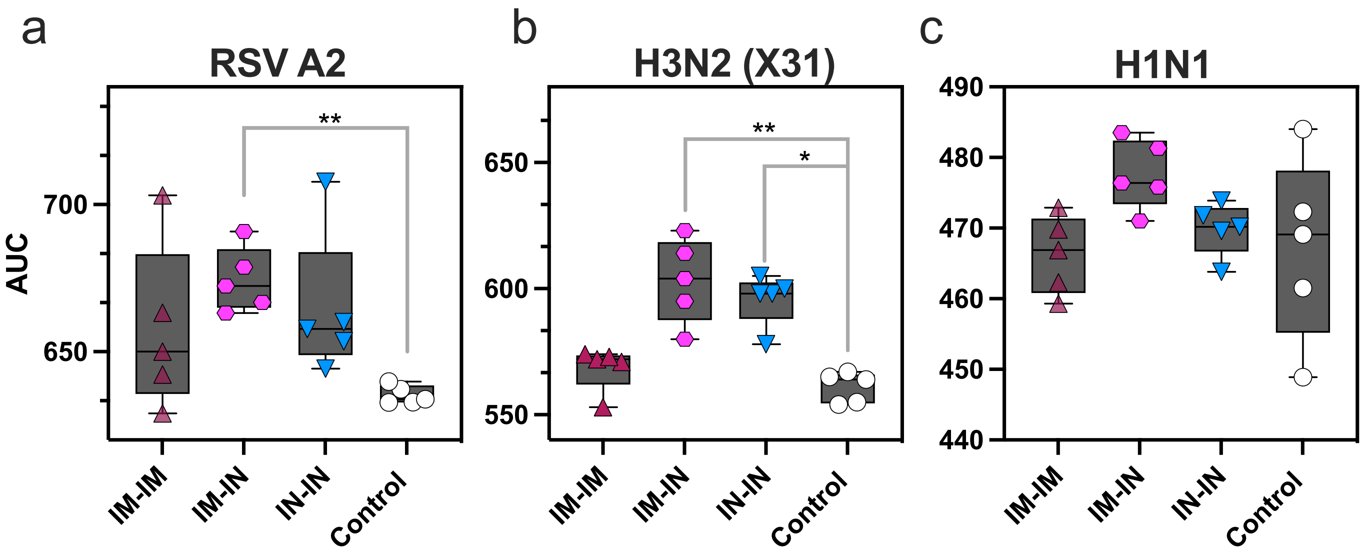

Supplementary Figure 5: Area under the curve (AUC) values for weight change upon challenge with RSV-A2 (a), H3N2 (b), or H1N1 (c). Individual values per mouse are represented as symbols. For all boxplots, whisker endings represent upper and lower extremes, the box bounds represent upper and lower quartiles, respectively, and the central line represents the group median. Values were analysed using nonparametric Kruskal-Wallis tests to assess for statistically significant differences between vaccine regimen groups, which are then expressed in p values (*=p<0.05, **=p<0.01).

Supplementary Figure 6

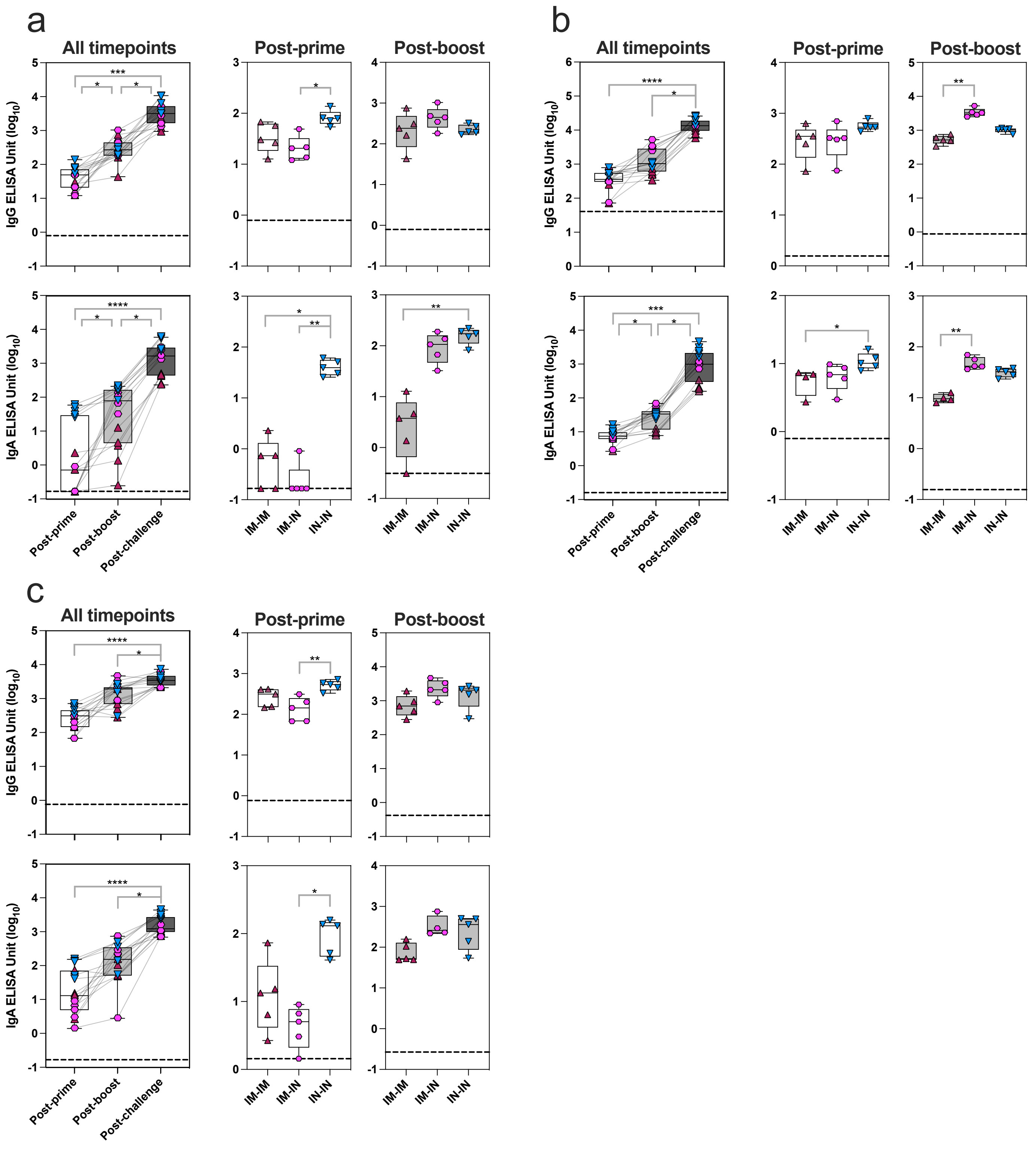

Supplementary Figure 6: (a) RSV A2-F-specific IgG and IgA levels in serum at all timepoints, and additionally post-prime and post-boost before challenge, as measured by standardised, indirect ELISAs in BALB/c mice. Values are displayed as EUs in log form. Median control values are displayed as dashed lines on graphs. Values were analysed using non-parametric Kruskal-Wallis tests to assess for statistically significant differences between vaccine regimen groups, and differences between timepoints for paired samples was determined through non-parametric Friedman test (*=p<0.05, ***=p<0.001, ****=p<0.0001). (b) H3N2 NP-specific IgG and IgA levels in serum at all time points, and additionally post-prime and post-boost before challenge, as measured by standardised, indirect ELISAs in BALB/c mice. (c) H1N1 NP-specific IgG and IgA levels in serum at all timepoints, and additionally post-prime and post-boost before challenge, as measured by standardised, indirect ELISAs in BALB/c mice. For all boxplots, whisker endings represent upper and lower extremes, the box bounds represent upper and lower quartiles, respectively, and the central line represents the group median.

Supplementary Figure 7

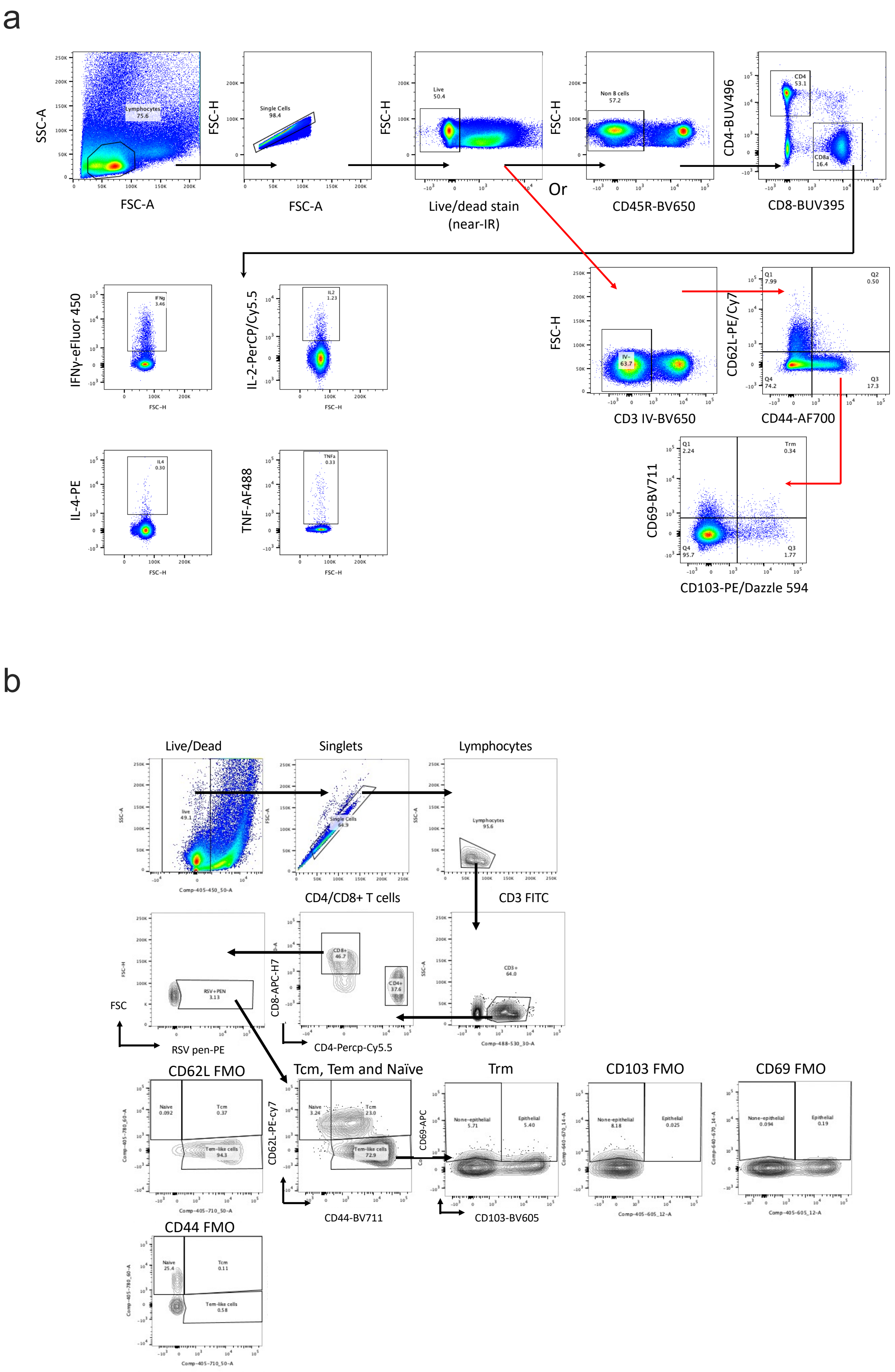

Supplementary Figure 7: (a) Intracellular staining flow gating strategy for assessment of cellular immunogenicity. For assessment of cytokine expression, CD45R was stained to exclude B cells from T cell gating. For assessment of lung T_{RM} populations, CD45R was not included in staining, and alternatively, CD3 antibody was injected into mice prior to cull to stain circulatory T cells that were excluded from the T_{RM} definition. (b) Flow gating strategy for infection studies.

Supplementary Table 1

a

	Kruskal-Wallis test significant P values					
	Serum IgG			Serum IgA		
	RSV A2-F	H1N1-NP	H1N1-M1	RSV A2-F	H1N1-NP	H1N1-M1
IM-IN>IM	0.0089	0.0017	0.0021	0.0013	0.0013	0.0029
IN-IN>IM	0.0049	0.0019	0.00323	0.0034	0.0061	0.0082
IM-IN>IN	0.0228	0.0128	0.0027			
IN-IN>IN	0.0126	0.0131	0.0401			
IM-IN>IM-IM				0.044	0.0489	

b

	Kruskal-Wallis test significant P values					
	H1N1-NP			RSV A2-F		
	NALT	BALF	LHS	NALT	BALF	LHS
IM-IN>IM-IM	0.0197		0.0255			0.0285
IN-IN>IM-IM	0.0134	0.032	0.0427	0.0433	0.0254	0.0102
IN>IM		0.0427		0.0184		
IN-IN>IM		0.0094	0.0093	0.0005	0.0094	0.0081
IM-IN>IM			0.0048	0.0051		0.0228

c

	Kruskal-Wallis test of significant P values	
	Serum IgG2a/IgG1 ratio	
	Influenza A NP	RSV F
IM>IN	0.014	0.0317
IM>IN-IN		0.0284

Supplementary Table 1: (a) Significant P values produced following Kruskal-Wallis test of levels of serum IgG and IgA specific to RSV A2-F, H1N1-NP and H1N1-M1 antigen in serum. P values demonstrate a statistically significant difference between two vaccination groups for a given antigen and antibody isotype. (b) Significant P values produced following Kruskal-Wallis test of levels of NALT, BALF and LHS IgA specific to RSV A2-F or H1N1-NP. P values demonstrate a statistically significant difference between two vaccination groups for a given antigen and respiratory fluid. (c) Significant P values produced following Kruskal-Wallis test of antigen-specific serum IgG2a/IgG1 ratios between groups. P values demonstrate a statistically significant difference between two vaccination groups.

Supplementary Table 2

a

	Kruskal-Wallis test significant P values			
	Days post-RSV A2 challenge			
	Day 4	Day 5	Day 6	Day 7
IM-IN>Control	0.0416	0.0327	0.0046	0.0067
IM-IM>Control				0.0385

b

	Kruskal-Wallis test significant P values			
	Days post-X-31 (H3N2) challenge			
	Day 3	Day 4	Day 5	Day 6
IM-IN>Control	0.0385	0.0031	0.0067	0.0066
IM-IN>IM-IM	0.0116			
IN-IN>Control		0.0234	0.0234	0.0233

Supplementary Table 2: (a) Significant P values produced following Kruskal-Wallis test of % weight change values between regimens at a given day post-RSV A2 challenge. (b) Significant P values produced following Kruskal-Wallis test of % weight change values between regimens at a given day post-X31 (H3N2) challenge.

Supplementary Table 3

a

	Kruskal-Wallis test significant P values							
	RSV-A2 challenge							
	IgG				IgA			
	Serum	NW	BALF	LHS	Serum	NW	BALF	LHS
IN-IN>IM-IM	0.0486	0.0327		0.0486	0.0027	0.0089	0.0016	0.0016

b

	Kruskal-Wallis test significant P values					
	H3N2 (X31) challenge					
	IgG			IgA		
	Serum	NW	BALF	Serum	NW	BALF
IN-IN>IM-IM	0.0016			0.0021	0.0056	0.0071

c

	Kruskal-Wallis test significant P values							
	H1N1 challenge							
	IgG				IgA			
	Serum	NW	BALF	LHS	Serum	NW	BALF	LHS
IN-IN>IM-IM			0.0486	0.014	0.0327	0.0352	0.0036	0.0012
IM-IN>IM-IM			0.0112			0.0119		

Supplementary Table 3: Significant P values produced following Kruskal-Wallis test of serum IgG and IgA levels specific to RSV A2-F (a), H1N1-NP (b) and H1N1-M1 (c) antigen in serum, NW, BALF and LHS following virus challenge. P values demonstrate a statistically significant difference between two vaccination groups for a given antigen and antibody isotype.

Supplementary Table 4

a

Pool for ICS	Pools for IFNg ELISpot	RSV(F)
Pool 1	Pool I	1 : MELLILKANAITTIL
		2 : ILKANAITTILTAVT
		3 : NAITTILTAVTFCFA
		4 : TILTAVTFCFASGQN
		5 : AVTFCFASGQNITEE
		6 : CFASGQNITEEFYQS
	Pool II	7 : GQNITEEFYQSTCSA
		8 : TEEFYQSTCSAVSKG
		9 : YQSTCSAVSKGYLSA
		10 : CSAVSKGYLSALRTG
		11 : SKGYLSALRTGWYTS
		12 : LSALRTGWYTSVITI
		13 : RTGWYTSVITIELSN
		14 : YTSVITIELSNIKEN
		15 : ITIELSNIKENKCNG
		16 : LSNIKENKCNGTDAK
		17 : KENKCNGTDAKVKLI
		18 : CNGTDAKVKLIKQEL
		19 : DAKVKLIKQELDKYK
		20 : KLIKQELDKYKNAVT
		21 : QELDKYKNAVTELQL
		22 : KYKNAVTELQLLMQS
		23 : AVTELQLLMQSTPAT
		24 : LQLLMQSTPATNNRA
		25 : MQSTPATNNRARREL
		26 : PATNNRARRELPRFM
		27 : NRARRELPRFMNYTL
		28 : RELPRFMNYTLNNAK
		29 : RFMNYTLNNAKKTNV
		30 : YTLNNAKKTNVTLSK
		31 : NAKKTNVTLSKKRKR
		32 : TNVTLSKKRKRRFLG
		33 : LSKKRKRRFLGFLLG
		34 : RKRRFLGFLLGVGSA
		35 : FLGFLLGVGSAIASG
		36 : LLGVGSAIASGVAVC
		37 : GSAIASGVAVCKVLH
		38 : ASGVAVCKVLHLEGE
		39 : AVCKVLHLEGEVNKI
		40 : VLHLEGEVNKIKSAL
		41 : EGEVNKIKSALLSTN
		42 : NKIKSALLSTNKAVV
		43 : SALLSTNKAVVSLSN
		44 : STNKAVVSLSNGVSV
		45 : AVVSLSNGVSVLTFK
		46 : LSNGVSVLTFKVLDL
		47 : VSVLTFKVLDLKNYI
		48 : TFKVLDLKNYIDKQL
		49 : LDLKNYIDKQLLPIL
		50 : NYIDKQLLPILNKQS
		51 : KQLLPILNKQSCSIS
		52 : PILNKQSCSISNIET
		53 : KQSCSISNIETVIEF
		54 : SISNIETVIEFQQKN
		55 : IETVIEFQQKNNRLL
		56 : IEFQQKNNRLLEITR
		57 : QKNNRLLEITREFSV
		58 : RLLEITREFSVNAGV
		59 : ITREFSVNAGVTTPV
		60 : FSVNAGVTTPVSTYM
		61 : AGVTTPVSTYMLTNS
		62 : TPVSTYMLTNSELLS
		63 : TYMLTNSELLSLIND
		64 : TNSELLSLINDMPIT
		65 : LLSLINDMPITNDQK
		66 : INDMPITNDQKKLMS
	Pool III	67 : PITNDQKKLMSNNVQ
		68 : DQKKLMSNNVQIVRQ
		69 : LMSNNVQIVRQQSYS
		70 : NVQIVRQQSYSIMCI
		71 : VRQQSYSIMCIIKEE
		72 : SYSIMCIIKEEVLAY
		73 : MCIIKEEVLAYVVQL
		74 : KEEVLAYVVQLPLYG
		75 : LAYVVQLPLYGVIDT
		76 : VQLPLYGVIDTPCWK
		77 : LYGVIDTPCWKLHTS
		78 : IDTPCWKLHTSPLCT
		79 : CWKLHTSPLCTTNTK
		80 : HTSPLCTTNTKEGSN
		81 : LCTTNTKEGSNICLT
		82 : NTKEGSNICLTRTDR
		83 : GSNICLTRTDRGWYC
		84 : CLTRTDRGWYCDNAG
		85 : TDRGWYCDNAGSVSF
		86 : WYCDNAGSVSFFPQA
		87 : NAGSVSFFPQAETCK
		88 : VSFFPQAETCKVQSN
		89 : PQAETCKVQSNRVFC
		90 : TCKVQSNRVFCDTMN
		91 : QSNRVFCDTMNSLTL
		92 : VFCDTMNSLTLPSEV
		93 : TMNSLTLPSEVNLCN
		94 : LTLPSEVNLCNVDIF
		95 : SEVNLCNVDIFNPKY
		96 : LCNVDIFNPKYDCKI
		97 : DIFNPKYDCKIMTSK
		98 : PKYDCKIMTSKTDVS
		99 : CKIMTSKTDVSSSVI
		100 : TSKTDVSSSVITSLG
		101 : DVSSSVITSLGAIVS
		102 : SVITSLGAIVSCYGK
		103 : SLGAIVSCYGKTKCT
		104 : IVSCYGKTKCTASNK
		105 : YGKTKCTASNKNRGI
		106 : KCTASNKNRGIIKTF
		107 : SNKNRGIIKTFSNGC
		108 : RGIIKTFSNGCDYVS
		109 : KTFSNGCDYVSNKGV
		110 : NGCDYVSNKGVDTVS
		111 : YVSNKGVDTVSVGNT
		112 : KGVDTVSVGNTLYYV
		113 : TVSVGNTLYYVNKQE
		114 : GNTLYYVNKQEGKSL
		115 : YYVNKQEGKSLYVKG
		116 : KQEGKSLYVKGEPII
		117 : KSLYVKGEPIINFYD
		118 : VKGEPIINFYDPLVF
		119 : PIINFYDPLVFPSDE
		120 : FYDPLVFPSDEFDAS
		121 : LVFPSDEFDASISQV
		122 : SDEFDASISQVNEKI
		123 : DASISQVNEKINQSL
		124 : SQVNEKINQSLAFIR
		125 : EKINQSLAFIRKSDE
		126 : INQSLAFIRKSDELL
	Pool IV	127 : SAIGGYIPEAPRDGQ
		128 : GYIPEAPRDGQAYVR
		129 : EAPRDGQAYVRKDGE
		130 : DGQAYVRKDGEWVLL
		131 : YVRKDGEWVLLSTFL
		132 : DGEWVLLSTFLGGLV
		133 : VLLSTFLGGLVPRGS
		134 : TFLGGLVPRGSHHHH
		135 : GLVPRGSHHHHHHSA
		136 : RGSHHHHHHSAWSHP
		137 : HHHHHSAWSHPQFEK

Pool for ICS	Pools for IFNg ELISpot	NP+M1
Pool 1	Pool I	1 : MASQGTKRSYEQMETDGDR
		2 : YEQMETDGDRQNATEIRASV
		3 : RQNATEIRASVGKMIDGIGR
		4 : VGKMIDGIGRFYIQMCTELK
		5 : FYIQMCTELKLSDYEGRLI
		6 : KLSDYEGRLIQNSLTIEKMV
		7 : IQNSLTIEKMVLSAFDERR
		8 : MVLSAFDERRNRYLEEHPSA
		9 : NRYLEEHPSAGKDPKKTGG
		10 : AGKDPKKTGGPIYRRVDGKW
		11 : PIYRRVDGKWMRELVLYDK
		12 : WMRELVLYDKEEIRRIWRQA
		13 : EEIRRIWRQANNGEDATAGL
		14 : NNGEDATAGLTHMMIWHSNL
		15 : THMMIWHSNLNDTTYQRTRA
		16 : NDTTYQRTRALVRTGMDPRM
		17 : LVRTGMDPRMCSLMQGSTL
		18 : MCSLMQGSTLPRRSGAAGAA
		19 : PRRSGAAGAAVKGIGTMVM
		20 : AVKGIGTMVMELIRMVKRGI
	Pool II	21 : ELIRMVKRGINDRNFWRG
		22 : GINDRNFWRGENGRKTRSAY
		23 : ENGRKTRSAYERMCNILKGK
		24 : ERMCNILKGKFQTAAQRAMV
		25 : FQTAAQRAMVDQVRESRNPG
		26 : DQVRESRNPGNAEIEDLIFL
		27 : NAEIEDLIFLARSALILRG
		28 : LARSALILRGSVAHKSCLPA
		29 : SVAHKSCLPACVYGPAVSSG
		30 : CVYGPAVSSGYDFEKEGYSL
		31 : YDFEKEGYSLVGIDPFKLL
		32 : LVGIDPFKLLQNSQVYSLIR
		33 : LQNSQVYSLIRPNENPAHK
		34 : IRPNENPAHKSQLVWMACH
		35 : KSQLVWMACHSAAFEDLRLL
		36 : SAAFEDLRLLSFIRGTKV
		37 : LLSFIRGTKVSPRGKLSTRG
		38 : SPRGKLSTRGVQIASNENM
		39 : GVQIASNENMDNMGSSTLEL
		40 : DNMGSSTLELRSGYWAIRTR
	Pool III	41 : RSGYWAIRTRSGGNTNQQRA
		42 : SGGNTNQQRASAGQISV
		43 : NQQRASAGQISVQPTFSVQR
		44 : SVQPTFSVQRNLPFEKSTVM
		45 : NLPFEKSTVMAAFTGNTEGR
		46 : AAFTGNTEGRTSDMRAEIIR
		47 : TSDMRAEIIRMMEGAKPEEV
		48 : MMEGAKPEEVSFRGRGVFEL
		49 : SFRGRGVFELSDEKATNPIV
		50 : SDEKATNPIVPSFEMSNEG
		51 : VPSFEMSNEGSYFFGDNA
		52 : EGSYFFGDNAEEYDNGGGPG
		53 : EEYDNGGGPGGGMSLLTEV
		54 : GGGMSLLTEVETYVLSIV
		55 : EVETYVLSIVPSGPLKAEIA
		56 : PSGPLKAEIAQRLEDVFAGK
		57 : AQRLEDVFAGKNTDLEALM
		58 : GKNTDLEALMEWLKTRPIL
		59 : MEWLKTRPILSPLTKGILGF
		60 : SPLTKGILGFVFTLTVPSER
	Pool IV	61 : VFTLTVPSERGLQRRRFV
		62 : ERGLQRRRFVQNALNGNG
		63 : FVQNALNGNGDPNNMDKAVK
		64 : DPNNMDKAVKLYRKLKREI
		65 : KLYRKLKREITFHGAKEIAL
		66 : TFHGAKEIALSYSAGALA
		67 : ALSYSAGALASCMGLIYNRM
		68 : SCMGLIYNRMGAVTTEVAFG
		69 : GAVTTEVAFGLVCATCEQIA
		70 : LVCATCEQIADSQHRSHRQM
		71 : DSQHRSHRQMVATTNPLIKH
		72 : VATTNPLIKHENRMVLA
		73 : IKHENRMVLASTTAKAMEQM
		74 : STTAKAMEQMAGSSEQAAEA
		75 : AGSSEQAAEAMEIASQARQM
		76 : MEIASQARQMVQAMRTVGTH
		77 : VQAMRTVGTHPSSSTGLR
		78 : THPSSSTGLRDDLLENLQTY
		79 : DDLLENLQTYQKRMGVQMQR
		80 : QKRMGVQMQRFK

Supplementary Table 4: (a) Overlapping peptides spanning preF RSV antigen and NP+M1 antigen. Peptides were pooled into one large pool per antigen for splenocyte and lung stimulation prior to ICS, or pooled into 4 separate pools per antigen for splenocyte stimulation for IFNγ ELISpot.
